# First principles study of chalcogen vacancy effect on the optoelectronic and photocatalytic properties of transition metal dichalcogenides monolayers

**DOI:** 10.1039/d6ra08882a

**Published:** 2026-11-03

**Authors:** Tahir Wahab, Antonio Cammarata, Tomas Polcar

**Affiliations:** a Department of Control Engineering, Faculty of Electrical Engineering, Czech Technical University in Prague Technicka 2 Prague 6 16627 Czech Republic wahabtah@fel.cvut.cz cammaant@fel.cvut.cz +420 224 91 8646 +420 224 35 5713; b Engineering Materials & nCATS, FEE, University of Southampton Southampton SO17 1BJ UK

## Abstract

In working conditions, chalcogen vacancies spontaneously occur in two-dimensional transition metal dichalcogenides (TMDCs) monolayers, affecting their optoelectronic and photocatalytic properties. To study how chalcogen vacancies affect such properties, we use quantum mechanical calculations considering prototypical MX_2_ (M = Mo, W, X = S and Se) TMDCs monolayers. Band structure calculations reveal that the introduction of vacancies produce electronic states about the Fermi level, hence resulting in the reduction of the band gap. Work function and electrostatic potential calculations show that the introduction of vacancies induce an asymmetry in the electrostatic potential, which may facilitate the charge separation; such feature is absent in a pristine monolayer. All the considered defective structures are capable of performing hydrogen evolution reaction, while co-catalyst is required to perform oxygen evolution reaction when used for water splitting. WS_2_ and WSe_2_ defective monolayers can serve as promising candidates for reducing CO_2_ into useful chemical products. The presented results show that vacancy-containing TMDCs monolayers own photocatalytic capabilities compared to the pristine counterparts, thus showing that defective TMD monolayers have prospective applications and should not be regarded as flawed products to be discarded. Finally, the results might constitute guidelines for the experimental synthesis of vacancy-engineered MX_2_ monolayers for optoelectronic devices and photocatalytic applications.

## Abstract 2

Chalcogen vacancies enable charge separation and photocatalytic activity in monolayer transition metal dichalcogenides.

## Graphical abstract

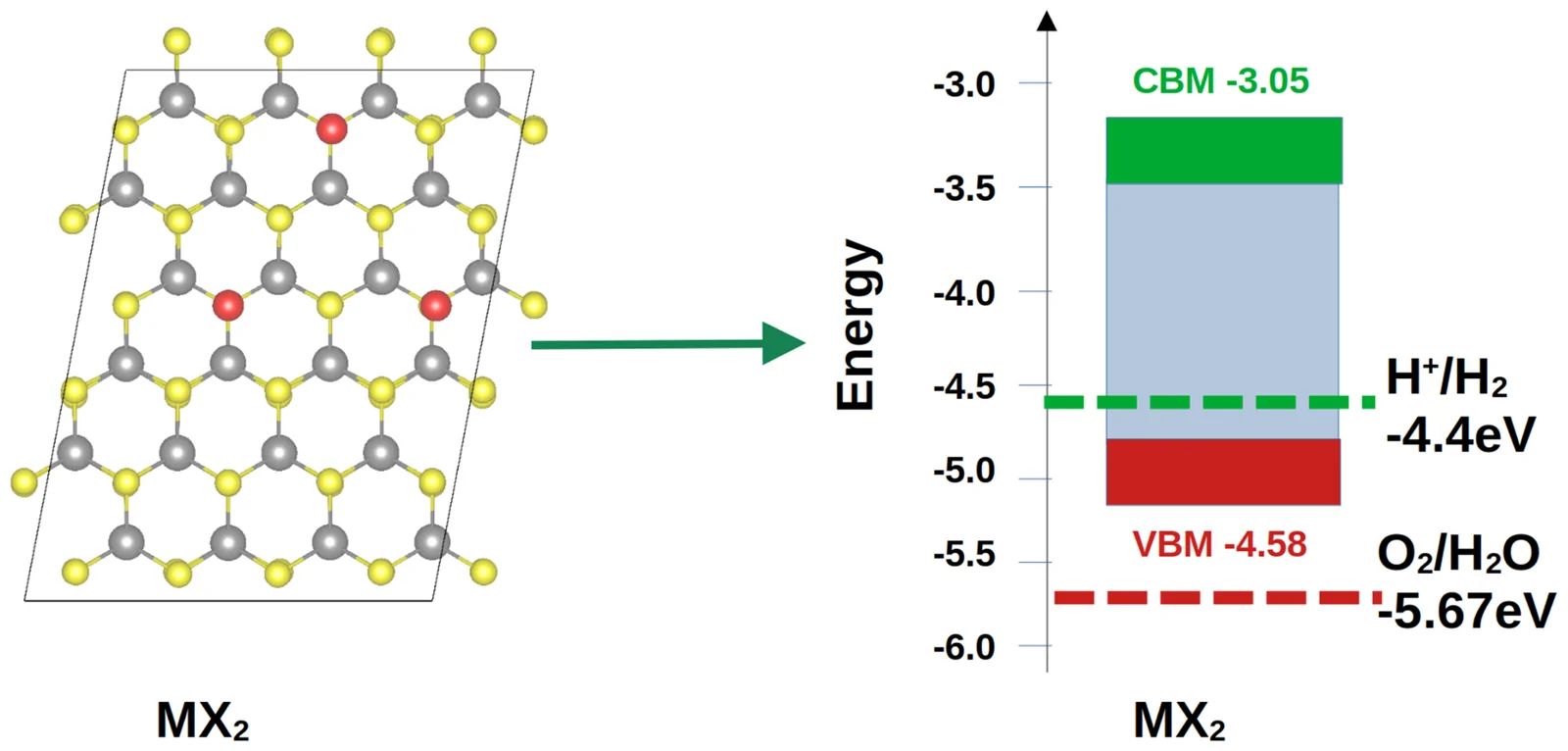

## 1 Introduction

Photocatalytic water splitting is an effective technique to convert solar light into molecular hydrogen and oxygen.^1–3^ The basic principle of photocatalytic water splitting is as follows: when a photon with energy equal to or greater than the band gap *E*_g_ of a photocatalytic semiconducting material is absorbed, electrons are excited from the valence band (VB) to the conduction band (CB); this generates a hole in the VB, and results in the formation of an exciton which is an electron–hole (e–h) pair.^3–6^ If the electron and hole in the pair are separated, they can migrate to the surface of the semiconductor, and can be used to split water molecules to perform hydrogen evolution reaction (HER) in the CB and oxygen evolution reaction (OER) in the VB.^3,6^ In order to perform the HER and OER, a semiconductor must satisfy the following conditions: (i) *E*_g_ must be greater than 1.23 eV and smaller than 3 eV, (ii) the gap width between the valence band maximum (VBM) and conduction band minimum (CBM) must straddle the water oxidation/reduction (redox) potential, and (iii) the semiconductor must exhibit excellent charge carrier separation and transport, *i.e.*, low recombination rate and efficient charge carrier separation.^4,7,8^ After the discovery of water splitting with TiO_2_ by Fujishima *et al.*,^9^ researchers are now considering a variety of photocatalytic materials, including metal oxides,^10,11^ metal sulfides,^12–14^ metal carbides,^15,16^ oxyhalides,^17,18^ oxynitrides,^19,20^ and recently transition metal dichalcogenides (TMDCs).^1,21–23^

Beyond HER, the photocatalytic reduction of CO_2_ into useful chemical products such as formic acid, formaldehyde, methanol, and methane is another technique to store sunlight in chemical bonds and mitigate the greenhouse emission.^24–27^ Similar to photocatalytic water splitting, a semiconductor that reduce CO_2_ to useful hydrocarbons should be low cost, highly stable, capable of absorbing visible light, excellent charge carrier separation and charge transfer.^4,28,29^ Various material studied for the photoreduction of CO_2_ includes metal oxides and mixed oxides,^30,31^ metal sulfides,^32–34^ polymeric materials,^35^ metal–organic framework (MOF),^36,37^ and TMDCs.^38–40^

TMDCs are materials with formula MX_2_, where M is a transition metal (TM) while X is a chalcogen (S, Se and Te), where the TM is sandwitched between two X layers.^41^ Because of the stability, large surface area, tunable and direct band gap nature, higher electrical conductivity, enhanced light absorption and strong excitonic effects, the two-dimensional group-VI TMDCs are excellent photocatalysts.^23,42–44^ However, the high carrier recombination rate of photogenerated charge carrier, low light harvesting capacity and slow reaction kinetics are the main reasons which hinder TMDCs to be efficient photocatalytic materials.^45^ Various techniques such as mechanical exfoliation,^46^ chemical exfoliation,^47^ hydrothermal intercalation,^48^ two-step expansion and intercalation^49^ and chemical vapour deposition (CVD)^46,50,51^ are used to exfoliate TMDCs from their bulk counterpart. During the synthesis, defects such as chalcogen vacancies are inevitable.^52^ It has been shown that pure basal planes are inactive in stable TMDCs, while anion vacancies can act as suitable active sites.^53,54^ First-principles calculations and investigations on samples obtained from mechanical exfoliation and CVD deposition showed that the presence of chalcogen vacancies is favourable in MoS_2_.^46,55^ Anion vacancies strongly influence the structural and optoelectronic properties, significantly enhancing the photocatalytic water splitting activity in these materials.^56–58^ The introduction of vacancies can then improve the photocatalytic ability by tuning the electronic band structure, increase the surface reactivity by increasing the number of active sites, enhance charge transport pathways, and influence the binding energy of all adsorbed species.^56,59,60^ The effect of chalcogen vacancies on the optoelectronic properties and photocatalytic activity of TMDC monolayer is well studied.^1,56,57^ It has been shown both experimentally as well as first principles calculations that activating basal plan in MoS_2_ can enhance the HER activity.^61,62^ Using first principles calculations, W. Chen *et al.* have identified the stable vacancy pair configurations in WS_2_ monolayer^63^ The affect of chalcogen vacancies on the optoelectronic properties have also been examined in MoSe_2_ and WSe_2_ monolayers.^64,65^ It has been proven experimentally that depending on the synthesis technique and growth conditions, the vacancy concentration in MX_2_ monolayer varies. Using scanning transmission electron microscopy (STEM) Hong *et al.* showed that the dominant defects type changes from chalcogen vacancy in chemical vapour deposition (CVD) and mechanical exfoliation to TM antisite in physical vapour deposition (PVD) samples.^46^ Depending on growth temperature, X-ray photoelectron spectroscopy (XPS) measurement on metal–organic CVD (MOCVD) grown MX_2_ monolayer showed that the chalcogen vacancy concentration increases from 0.9 at% to 3.6 at%^66^ which shows that the vacancy concentration depends on the growth kinetics and temperature. However most of the studies consider either arbitrary vacancy concentration or the one got from the experiment. While we instead of aiming to reproduce a particular vacancy concentration from experimental sample or a random concentration, use single and consistent method for all the four MX_2_ monolayers which enable us to identify the vacancy concentration independent of the growth technique, and thus can compare the trends across all the four MX_2_ monolayers. The novelty of our investigations lies in the use of cluster expansion approximation of the interactions as implemented in the Alloy Theoretic Automated Toolkit (atat)^67^ coupled with the Vienna *ab initio* simulation package (vasp)^68^ to find the vacancy concentration which realises the minimum of the energy convex hull. In this way, we identify the defective structure which is favourable to form within the convex-hull analysis, and used these consistent set of structures in our further investigations. The vacancy concentration in the experiment will depend on the chemical potential, growth conditions, temperature, and the kinetic effects; these effects are not included in the convex-hull construction and constitute the subject of a dedicated study which is out of the scope of the present work. In this framework, we here investigate the effect of chalcogen vacancies on the optoelectronic and photocatalytic performance of prototypical MX_2_ monolayers. The introduction of vacancies result in the reduction of the band gap by producing electronic states within the band gap. The optical absorption coefficient show a red shift in MoX_2_ monolayer, while all the structures are capable of performing hydrogen evolution reaction, and photocatalytic reduction of CO_2_ into useful products.

## 2 Calculation method

The reference for our computational models is the geometry of the prototypical MX_2_ monolayer, with M = Mo, W and X = S and Se, which we already used to study the optical properties and photocatalytic activity of TMDC-based van der Waals heterostructures^69^ (Fig. 1). We use the cluster expansion approximation of the interactions as implemented in the Alloy Theoretic Automated Toolkit (atat)^67^ coupled with the Vienna *ab initio* simulation package (vasp)^68^ to find the vacancy concentration which realises the minimum of the energy convex hull. This serves us to identify the defective structure which is the most energetically favoured to form (Fig. 2). We perform density functional theory calculations by using the Perdew–Burke–Ernzerhof (PBE)^70^ version of the generalized gradient approximation. The lattice parameters of the defective structures are large enough (see SI, section “Optimised geometries”) to justify the use of only the *Γ*-point to sample the Brillioun zone. The model geometries are relaxed by using a tolerance of 10^−8^ eV and 10^−3^ eV per Ĺ on the self consistent field procedure and the forces, respectively. A convex hull^71^ is calculated as a function of the vacancy concentration for each selected MX_2_ chemical composition; the structure realising the minimum in the convex hull is then selected for further investigation. All the first principles calculations are performed using the vasp software employing the projector-augmented wave^72^ method, and using a minimum of 450 eV for the plane wave cutoff. To account for the long range van der Waals interactions, Grimme's (DFT-D2)^73^ correction is used. To investigate the thermal stability of the selected structures, we perform *ab initio* molecular dynamics simulations^74^ using Nosé–Hoover thermostat^75^ with temperature equal to 300 K with a time step of 1 fs and a total time window of 8 ps. The dynamical stability of all the structures is then examined by calculating the phonon dispersion by means of the phonopy software.^76,77^ We calculate the electronic band structure of the selected geometries by using the PBE^78^ and Heyd–Scuseria–Ernzerhof (HSE06) functionals.^79^ Phonon dispersions and electronic band structures are calculated along the standard^80^ piecewise linear path *Γ*–*M*–*K*–*Γ* in the irreducible Brillioun zone. To examine the variation of the electrostatic potential along the *c*-axis and the charge redistribution, we perform a planar average electrostatic potential calculations along the same axis at the HSE06 level.

**Fig. 1:**
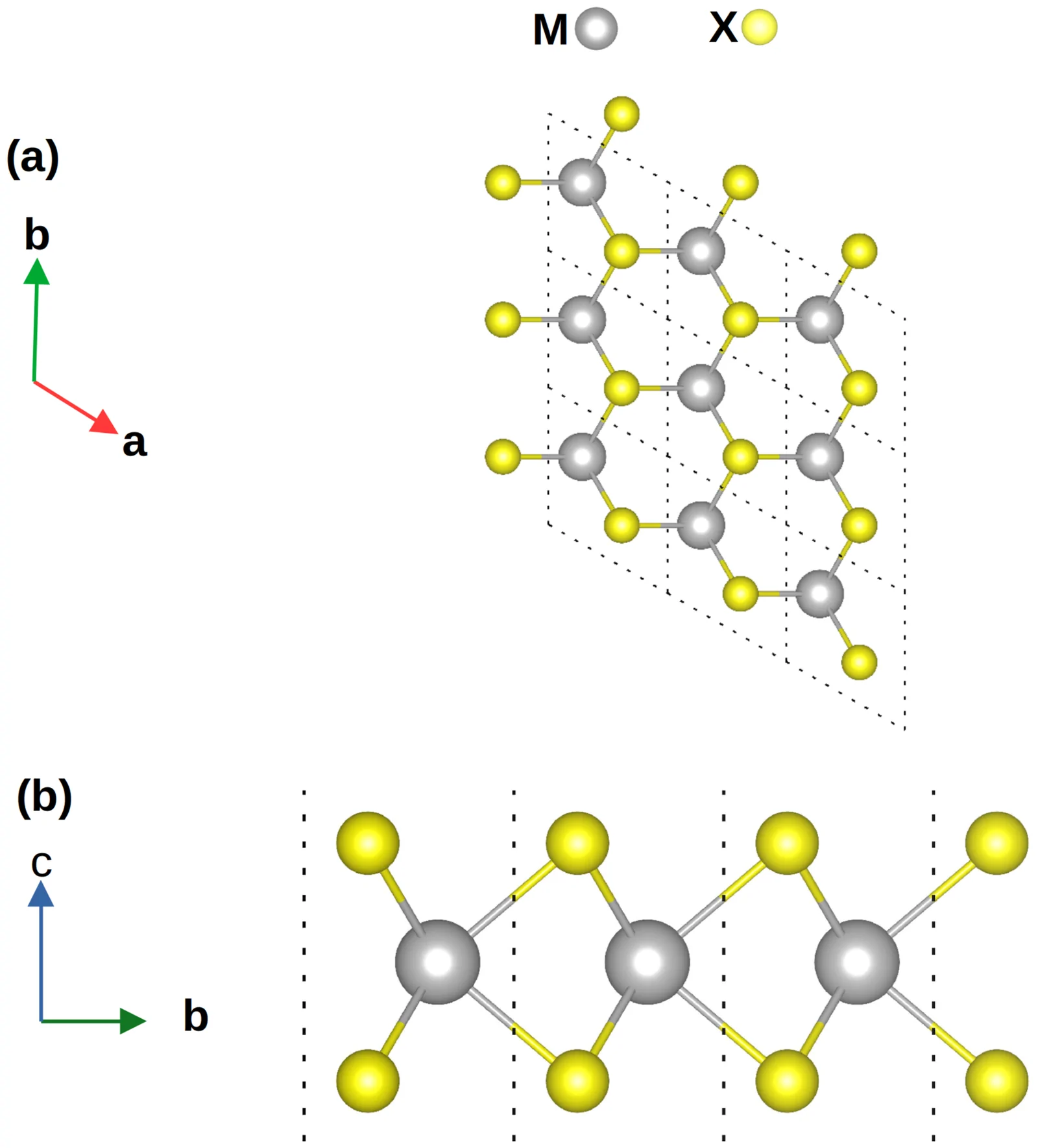
(a) Top and (b) side view of pristine MX_2_ monolayer model geometry; the *a*, *b*, *c* lattice parameters are reported for reference. The dashed lines indicate the limits of the unit cell.

**Fig. 2:**
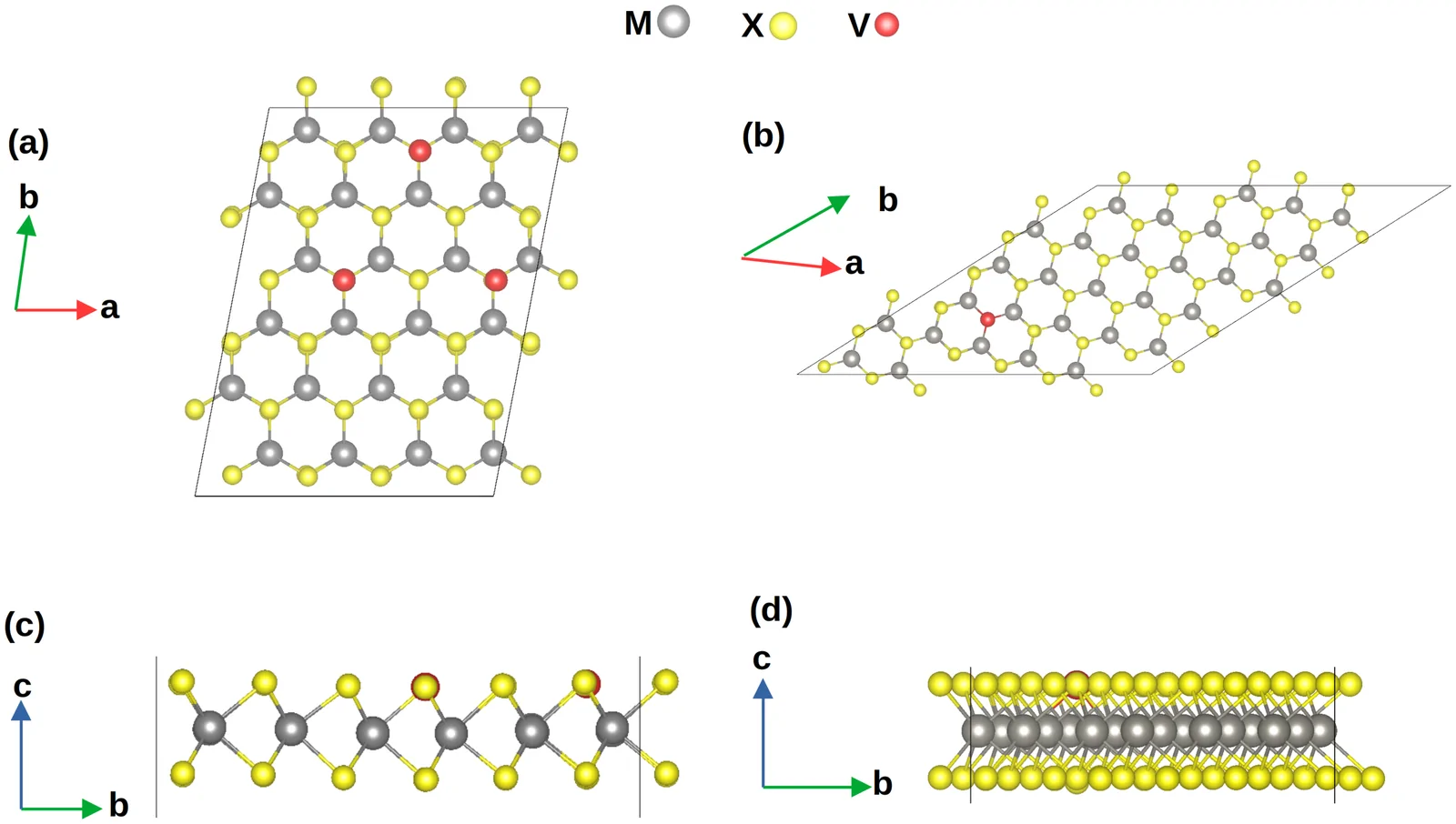
Example of defective MX_2_ monolayer: (a and b) top and (c and d) side views showing the presence of a vacancy (indicated with a red sphere and labeled as “V”) at the chalcogen site.

## 3 Results and discussion

As mentioned in the previous section, we already investigated the optoelectronic properties and photocatalytic water spliting performance of pristine MX_2_ monolayers;^69^ we showed that all the monolayers straddle the water redox potential, and hence are capable of water splitting due to their favourable band gap and band alignment for hydrogen and oxygen evolution reaction. As we know that defects such as chalcogen vacancies are inevitable, the goal here is to examine the effect of chalcogen vacancies on the optoelectronic properties and the photocatalytic performance.

The first step is to determine which chalcogen vacancy concentration is preferred in MX_2_ monolayers. To this aim, we use atat to comprehensively enumerate all the vacancy configurations on the chalcogen sublattice and construct the convex hull of the formation energy as a function of the vacancy concentration. In atat, the convex hull is computed as part of the ground-state search: candidate superstructures are enumerated, their formation energies are predicted from the current cluster expansion, and the lower convex hull in energy-composition space identifies which structures are ground states; the fit is improved iteratively by running *ab initio* calculations on structures near or below the hull until no new ground states appear. Energies and hull vertices are reported per atom relative to reference states. For a binary alloy there is only one independent composition variable (*i.e.* the anion vacancy), so the convex hull is constructed in the two-dimensional plane of formation energy per atom *versus* composition. The formation energy of each candidate structure is measured relative to the pure-element reference states, so the hull directly expresses how much more stable a configuration is than a simple physical mixture of the end members. Candidate structures are generated by exhaustively enumerating all superstructures of the parent lattice containing up to *n* atoms per unit cell, evaluating their energies with the current cluster expansion, and the limit *n* is raised automatically as the search progresses. The lower convex hull of these points is then computed: the structures lying on the hull are the ground states, *i.e.* the lowest-energy configuration at their composition, while the hull's line segments between adjacent vertices are tie lines indicating that intermediate compositions achieve minimum energy by phase-separating into a mixture of the two neighboring ground states. Whenever the cluster expansion predicts a structure lying below the current hull, that structure is flagged and its energy is recalculated from first principles, the fit and hull are updated accordingly, and this self-correcting loop continues until no new ground states are found. Following this procedure, the concentrations realising the lowest energy in the convex hull are found to be 6.25% for MoS_2_, MoSe_2_, WSe_2_ defective systems, respectively, while 1.56% for the WS_2_ system (Fig. 3); accordingly, the size of the supercell used in our models has been chosen to represent the corresponding concentrations for each considered system. Each vacancy is surrounded by three cations coordinating X anions in their first coordination shell (Fig. 2). We do not observe a significant change in the M–X bond lengths in the defective structures when compared to the pristine geometries. This suggests that no significant local strains are occurring about the vacancies and, correspondingly, the stability of the structure should not be affected.

**Fig. 3:**
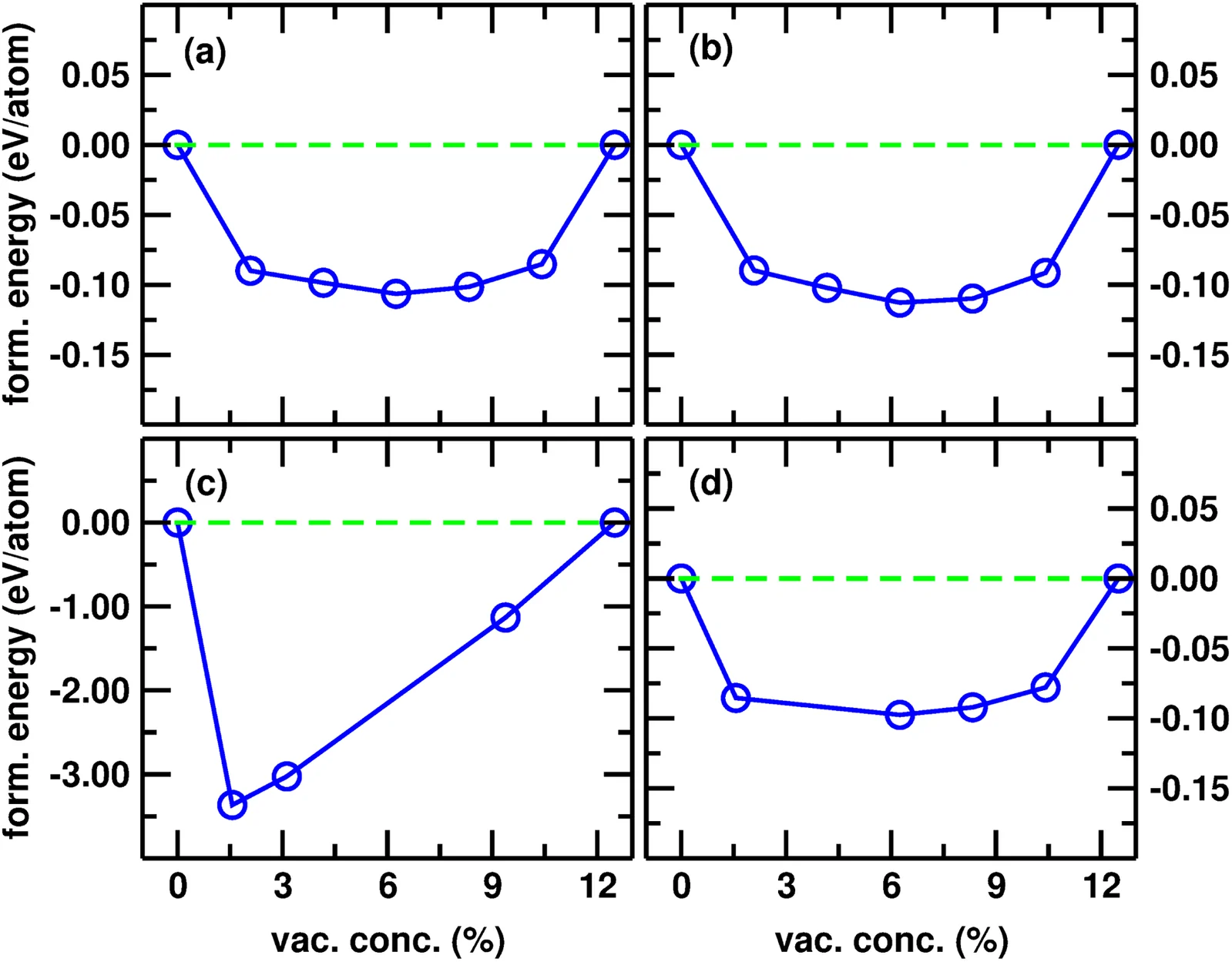
Convex hull calculated for (a) MoS_2_, (b) MoSe_2_, (c) WS_2_, and (d) WSe_2_ systems at variable anion vacancy concentration. The energy of the structures at the endpoints are taken as a reference and set to zero by atat (dashed line).

We calculate the formation energies according to the equation^81^*E*_f_ = *E*^tot^_vac_ – *E*^tot^_pr_ + *nµ*_X_, where *E*^tot^_vac_ and *E*^tot^_pr_ represent the total energies of relaxed defective and pristine structure respectively, while *n* is the number of vacancies; *n* = 3 in the case of MoX_2_ and WSe_2_, while *n* = 1 for WS_2_. *µ*_X_ is the chemical potential of the removed chalcogen atom, *i.e.*, the energy per atom of the bulk elemental phase, which for sulphur is the α-S_8_ allotrope with crystallographic space group *Fddd* (group no. 70), and for selenium is the trigonal Se with space group *P*3_1_21 (group no. 152). The formation energies per vacancy for MoS_2_, MoSe_2_, WS_2_, and WSe_2_ are found to be 2.83, 2.73, 3.00 and 2.86 eV, respectively. The reported formation energies are calculated under the chalcogen-rich condition^82,83^ and thus represent an upper bound.

To assess the structural stability of the selected systems, we first calculate the phonon dispersion (Fig. 4) along the standard path in the Brillouin zone (see Section 2). The phonon band structure is free of imaginary frequencies except for a small pocket near the *Γ* point in the acoustic branch, which is known to be a numerical artifact in 2D transition metal dichalcogenides as mentioned by Fal'ko *et al.*^84^ To confirm that such imaginary frequencies are an artifact and that the structure is dynamically stable at operating temperature, we perform AIMD simulations at 300 K. Fig. 5 shows the fluctuation in cation–anion M–X distance Δ*d*_M–X_(*t*), temperature and total energy as a function of the time, respectively (see also SI, section “*Ab Initio* Molecular Dynamics Simulations” for further details). The fluctuation in bond length is defined as Δ*d*_M–X_(*t*) = *d*_M–X_(*t*) − 〈*d*_M–X_〉, where *d*_M–X_(*t*) is the M–X bond length at time step t, and 〈*d*_M–X_〉 is the time average *M*_X_ bond length over the entire steps. We see that the fluctuation in M–X bond length oscillates about the mean value over the entire trajectory (Table 1), which indicates that no bond breaking occurs and the structure is thermodynamically stable. For all the systems, we observe the temperature fluctuates about the target value of 300 K, while the total energy exhibits small oscillations about the average value. Therefore, phonon band and AIMD simulations confirm that the defective structures containing vacancies are dynamically and thermally stable, and it is then worthy to investigate their optical and photocatalytic response.

**Fig. 4:**
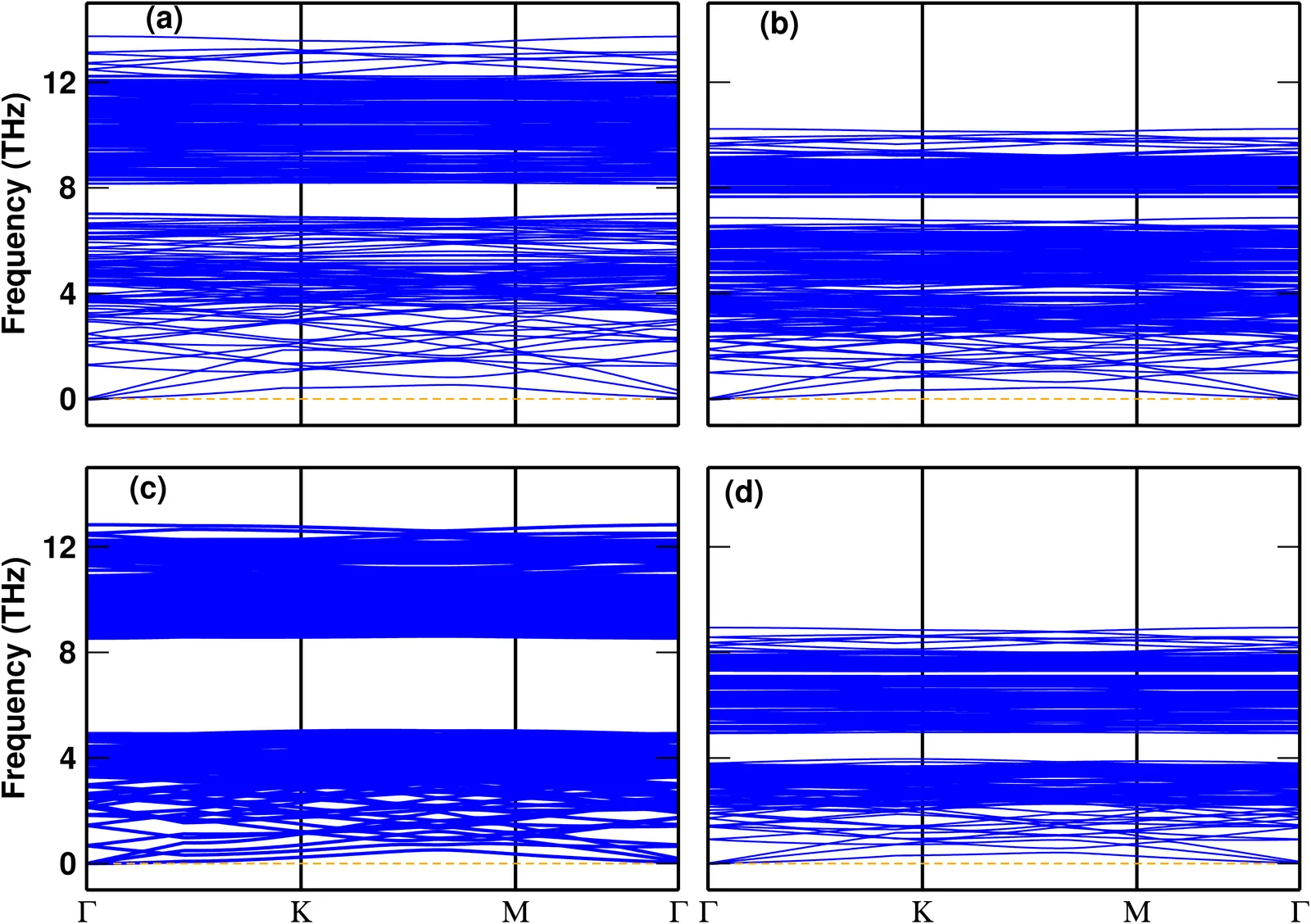
Phonon band dispersion along a piecewise linear path in the irreducible Brillioun zone of (a) MoS_2_, (b) MoSe_2_, (c) WS_2_, and (d) WSe_2_ systems. The dashed orange horizontal line indicates the position of zero frequency.

**Fig. 5:**
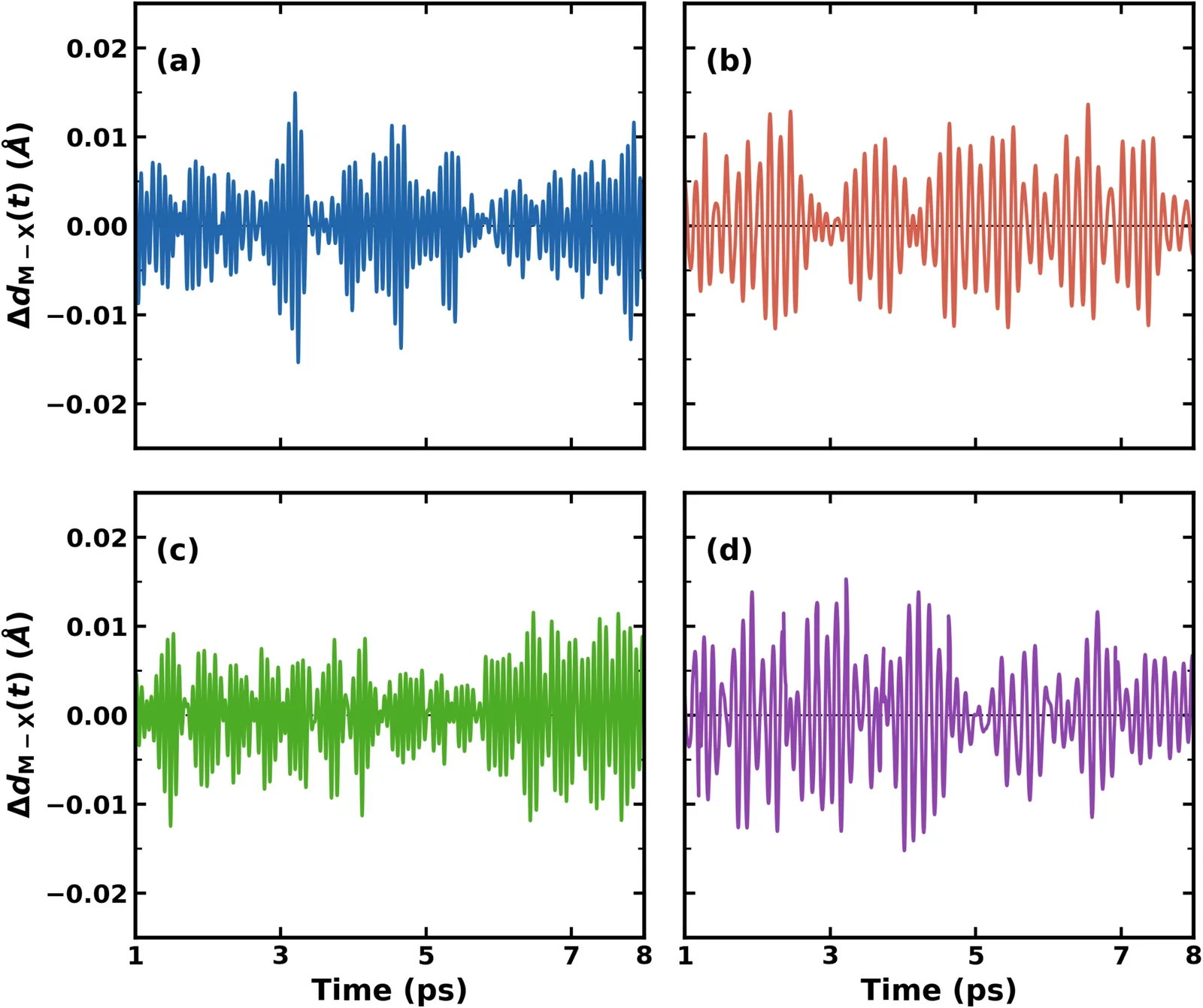
M–X bond length as a function of the simulation time of the (a) MoS_2_, (b) MoSe_2_, (c) WS_2_, and (d) WSe_2_ systems.

**Table 1:** Average M–X bond lengths with the corresponding standard deviation about X vacancies (Å). *P* and *V* indicate the values for the pristine and defective monolayer, respectively; standard deviation for WS_2_ is zero as all the W–S bond lengths have equal values. No standard deviation is reported for the pristine monolayers because the M cation coordinates X neighbouring atoms in a regular polyhedron. The values of the pristine monolayers are taken from ref. 69

Structure	Bond length *P*/*V*
MoS_2_	2.41/2.40 ± 0.01
MoSe_2_	2.54/2.53 ± 0.01
WS_2_	2.41/2.41 ± 0.00
WSe_2_	2.54/2.56 ± 0.01

We then continue our analysis by calculating the electronic band structure at the PBE (see SI, section “Electronic band structures”) and HSE06 (Fig. 6) theory level. According to the PBE calculations, only WSe_2_ exhibits a direct band gap, both VBM and CBM lying at the *K* point, while all the remaining structures exhibit indirect band gap. We note that the introduction of chalcogen vacancies introduce states within the band gap which are not present in the pristine monolayer; as a result, the band gap of the defective structures is smaller compared to the gap width found in the pristine ones. According to HSE06 calculations (Fig. 6), WS_2_ and WSe_2_ exhibit a direct band gap while MoS_2_ and MoSe_2_ exhibit indirect band gap. The HSE06 calculated band gap values for MoS_2_, MoSe_2_, WS_2_, and WSe_2_ are 1.25, 1.31, 1.85, and 1.53 eV respectively. We find that (Fig. 6) the major contribution to both VBM and CBM comes from the transition metal d-orbitals^85^ around the vacancy, while the chalcogen atom near the vacancy does not contribute to the energy levels about the band gap in a significant way. However we can also see some minor contribution by the chalcogen: in the case of MoS_2_ and MoSe_2_, the chalcogen has a minor contribution to VBM, while in the case of WS_2_ it has a minor contribution to CBM. The introduction of spin–orbit coupling (SOC) in the system Hamiltonian results in the splitting of VBM and CBM for W-based TMDCs.^86–88^ We then calculate the band structure of defective WS_2_ and WSe_2_ systems by incorporating the SOC effects at the PBE level (see SI, section “Electronic band structures”). We project the spin of each state from PBE + SOC band structure on the *x*, *y* and *z*-axis in order to the examine the Rashba-type spin splitting. The PBE + SOC spin projected bands indicate that the spin in the VBM, CBM, and in the states induced by the vacancy is polarized out of plane, *i.e.*, predominantly of Zeeman-type, and thus we do not observe Rashba-type spin splitting in VBM and CBM (see SI, section “Electronic band structures”). We observe that the introduction of SOC results in lowering the CBM by −199 meV for WS_2_, and −202 meV for WSe_2_. However, despite the shift, in both the cases CBM still lies above the reduction potential of water and the two W-based structures are capable of performing HER. We are aware of the limitations of the PBE functional in the estimation of the band edges;^89–91^ however, the magnitude of the shift in CBM for both WS_2_ and WSe_2_ by including the SOC at HSE06 level is nearly 380 meV,^92^ which is much smaller when compared to the difference between the CBM from H^+^/H_2_ and CO_2_ reduction potentials. Therefore, our conclusion about HER and CO_2_ reduction abilities of the two W-based structures still holds.

**Fig. 6:**
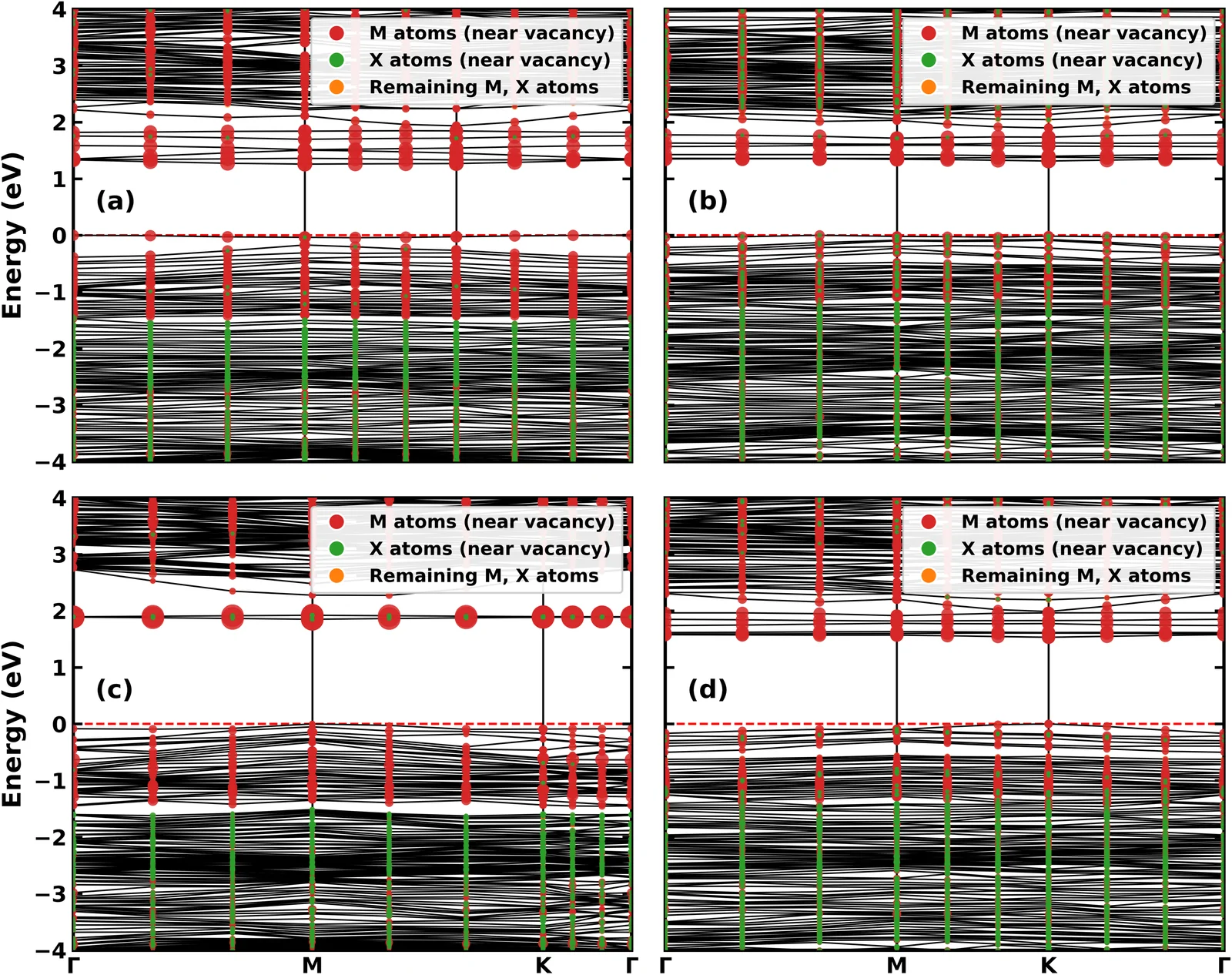
Atom-resolved band structure of (a) MoS_2_, (b) MoSe_2_, (c) WS_2_, and (d) WSe_2_ defective systems, calculated at the HSE06 theory level. The Fermi level has been set to 0 eV and is indicated by a dashed line.

In order to calculate the electrostatic potential asymmetry induced by the presence of vacancies, we calculate the planar average electrostatic potential along the *c*-axis (Fig. 7). As compared to the layer bearing vacancies, the electrostatic potential is more negative on the pristine chalcogen layer; this can be ascribed to the fact that the introduction of vacancies at the chalcogen site results in the reduction of local electron density and hence in a less negative electrostatic potential on the vacancy side. We then calculate the work function *ϕ* as

1 *ϕ* = *E*_vac_ − *E*_F_

where *E*_F_ and *E*_vac_ represent the Fermi and the vacuum level, respectively, the latter obtained from the plateau of the electrostatic potential in correspondence to the vacuum slab. The vacuum level on the vacancy side is larger as compared to the pristine anion side; this produces two different work function values, one for each side of the monolayer.^69,93^ In all the structures, *ϕ* on the vacancy side is higher than the value on the pristine side, with a difference δ*ϕ* of 21, 83, 19 and 89 meV for MoS_2_, MoSe_2_, WS_2_, and WSe_2_, respectively. It can be seen that δ*ϕ* in the Se based structure is larger as compared to the S based structures; this correlates with the greater polarizability of Se compared to S, and hence a prominent asymmetry in the surface potential. In a pristine MX_2_ monolayer, due to the symmetrical distribution of the chalcogen atoms with respect to the cation layer, the electrostatic potential is symmetric with respect to the same layer; as a consequence, *ϕ* assumes the same value on both sides of the monolayer and there is no built-in electric field which can separate the photogenerated charge carriers. Instead, we can see that the introduction of vacancies on one of the two X layers results in an asymmetry of the electrostatic potential together with a δ*ϕ*, which determines a built-in electric field that may facilitates charge separation, and hence can be considered as promising for reducing charge recombination. Compared to the MS_2_ systems, the larger values of δ*ϕ* in the MSe_2_ systems suggest a strong built-in electric field which can be more favorable for the photogenerated charge carriers separation. The trends in the *ϕ*, δ*ϕ* and the electrostatic potentials is similar to what has been observed in a previous work,^85^ where the difference of the reported values is due to the different concentration of vacancies we have in the present work.

**Fig. 7:**
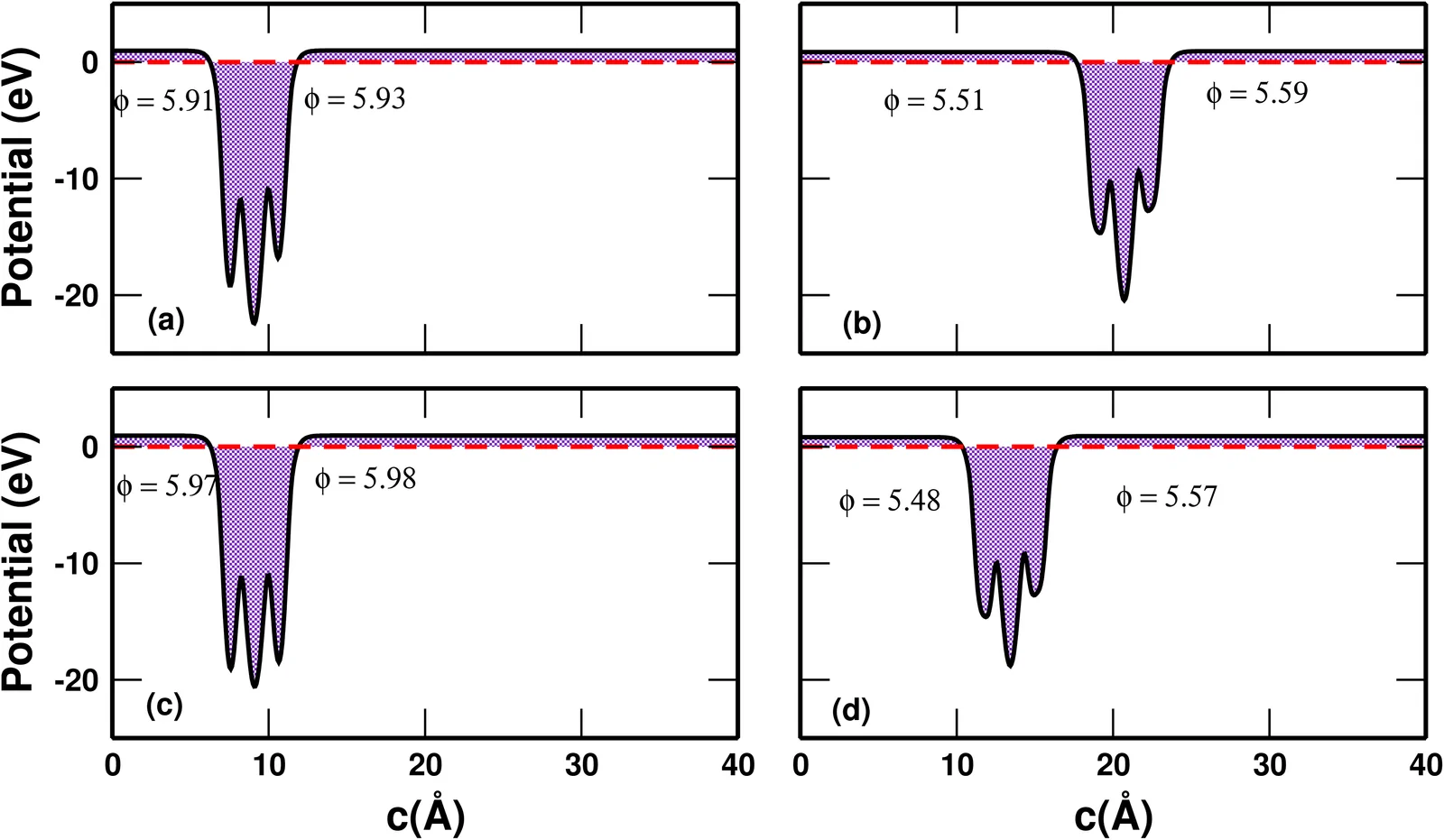
Planar-average electrostatic potential along the *c* axis of (a) MoS_2_, (b) MoSe_2_, (c) WS_2_, and (d) WSe_2_ defective systems, calculated at the HSE06 theory level.

We then continue our investigation by performing Bader charge analysis,^94^ in order to calculate the charge redistribution induced by the introduction of vacancies. The vacancy-induced charge transfer is calculated as follows. We consider the chalcogen and the transition metal atoms surrounding the vacancy and calculate the average charge *q*_vac_; we also consider the same chalcogen and transition metal atoms in the corresponding pristine structure and calculate the average charge *q*_pr_. The vacancy-induced charge transfer is then defined as *δ*_q_ = *q*_pr_ – *q*_vac_,^95^ where negative (positive) *δ*_q_ values indicate an excess (lack) of electrons in the defective structure. The calculated *δ*_q_ values on the TM around the vacancies are −0.37, −0.18, −0.30 and −0.30 |*e*| for MoS_2_, MoSe_2_, WS_2_ and WSe_2_, respectively, while on the chalcogen are 0.08, 0.13, 0.01, and 0.17 |*e*| for MoS_2_, MoSe_2_, WS_2_ and WSe_2_, respectively. This analysis shows that the vacancy induces a redistribution of charge on the atoms surrounding it; this results in a change of the local electrostatic potential, which may thus favour charge separation across the layer.

We then calculate the optical absorption coefficient *α*^96^ in the [0, 6] eV energy range (Fig. 8). For MoS_2_, the first absorption peak lies within the infrared region, while for MoSe_2_ the first absorption peak lies near the visible region. Concerning WS_2_, the absorption starts in the visible region, while the first absorption peak lies in the UV range, while for WSe_2_ the first absorption peak lies within the visible range. It can be seen that, as compared to the pristine case, all the MoX_2_ monolayers containing vacancies show a red shift in the first absorption peak.^69^ In fact, as observed above, the presence of anion vacancies results in the introduction of electronic states within the band gap (Fig. 6), which facilitates the absorption of light with lower energy than that required in the case of pristine monolayers,^69^ including infrared radiation.

**Fig. 8:**
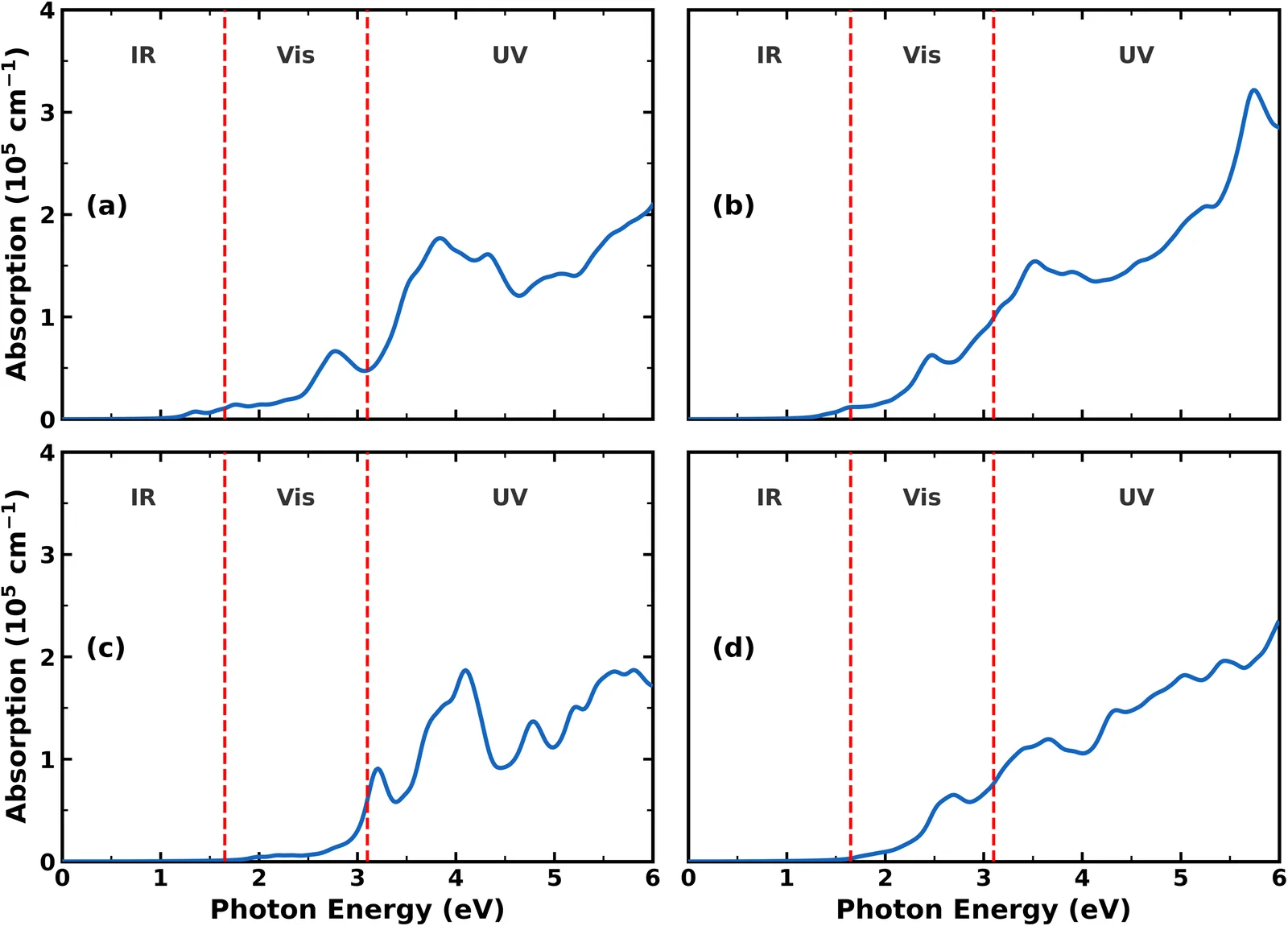
Calculated optical absorption spectrum of (a) MoS_2_, (b) MoSe_2_, (c) WS_2_, and (d) WSe_2_ defective systems.

In order to check the photocatalytic water splitting activity, we compare the HSE06 calculated VBM and CBM with water redox potential (Fig. 9a). We here recall that a semiconductor must satisfy two conditions to split the water molecule: the band gap should be greater than 1.23 eV, and the band edges must straddle the water redox potential.^97^ The HSE06 calculated band gap value of all the considered structure is greater than 1.23 eV. Also, it can be seen that the CBM of all the monolayers lie above the potential required for HER, suggesting that, under visible light illumination, the electrons in the CBM have enough energy to reduce hydrogen from the water molecule. However, the VBM also lies above the potential required for the OER, this suggesting a limited capability of performing OER under sun light.

**Fig. 9:**
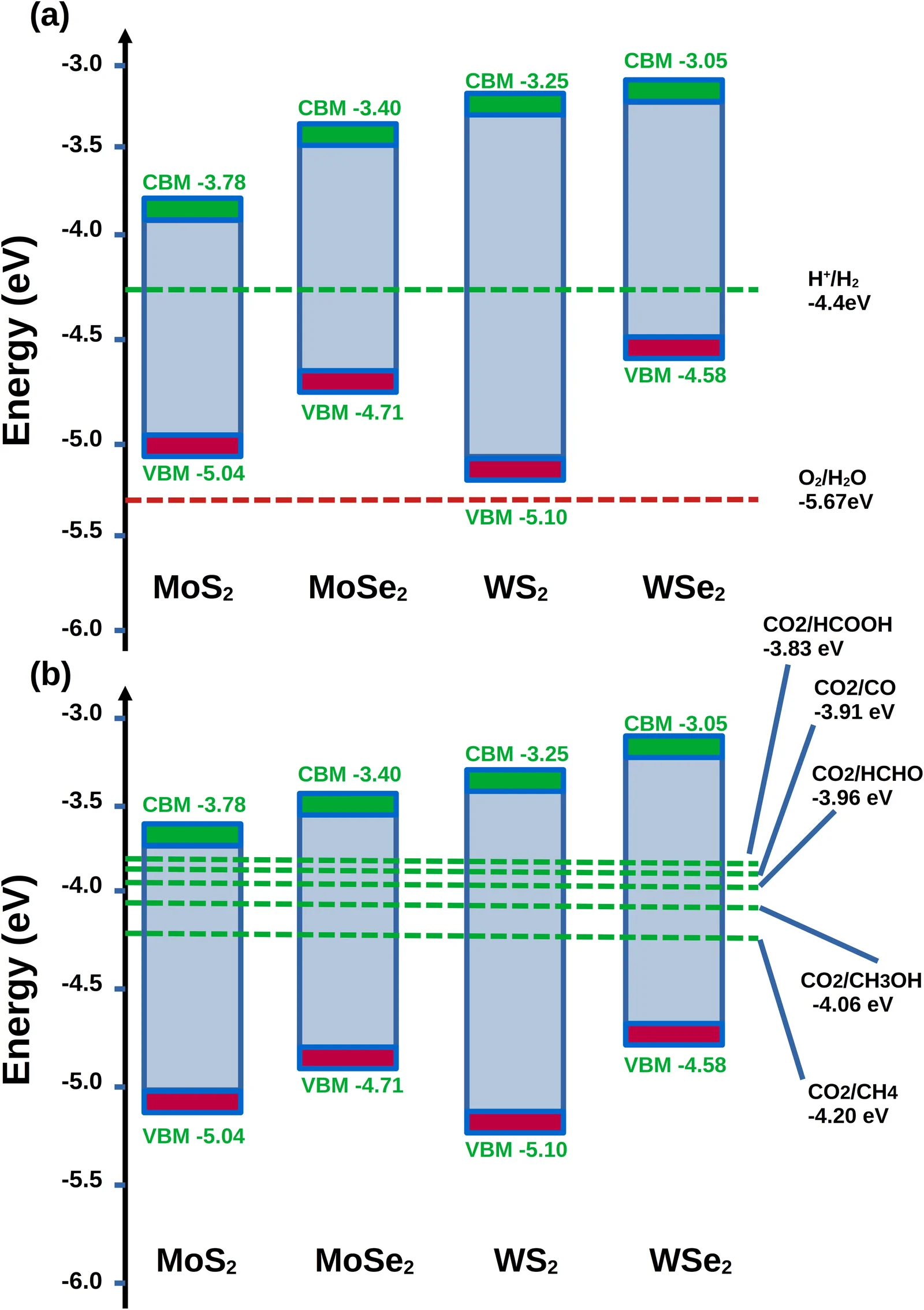
CBM and VBM of all MX_2_ structure in comparison (a) water redox potential and (b) CO_2_ reduction potential.

Next we examine the CO_2_ reduction capability of these structures by comparing the HSE06 calculated CBM with CO_2_ reduction products on the vacuum scale (Fig. 9b). To compare the HSE06 calculated band edges and the CO_2_ reduction potentials,^31^ we consider the reduction potential for the CO_2_ reported *versus* normal hydrogen electrode (NHE) related to the vacuum scale by using the relation *E*_vac_ = −*E*_NHE_ − *E*_0_, where *E*_vac_ represents the redox potential referenced to free electron in vacuum *E*_NHE_ against the NHE, and *E*_0_ = 4.44 eV is the absolute potential of the standard hydrogen electrode.^98,99^ The equations representing the reduction half reactions and converting CO_2_ in carbon products^31,100,101^ are as follows

2 CO_2_ + 2H^+^ + 2e^−^ → HCOOH

3 CO_2_ + 2H^+^ + 2e^−^ → CO + H_2_O

4 CO_2_ + 4H^+^ + 4e^−^ → HCHO + H_2_O

5 CO_2_ + 6H^+^ + 6e^−^ → CH_3_OH + H_2_O

6 CO_2_ + 8H^+^ + 8e^−^ → CH_4_ + 2H_2_O.

At pH = 7 the potential required for the half reduction reaction to convert CO_2_ to formic acid (HCOOH), carbon monoxide (CO), formaldehyde (HCHO), methanol (CH_3_OH) and methane (CH_4_) *versus* NHE are −0.61, −0.53, −0.48, −0.38 and −0.24 respectively, which after conversion becomes −3.83, −3.91, −3.96, −4.06 and −4.20 eV with respect to the vacuum level respectively, while the potential requires for OER is 0.82 V *versus* NHE, while −5.26 eV on the vacuum scale.^31,101^ We know that the minimum band gap requirement for a semiconductor to split water is 1.23 eV, which is the difference of potential between HER and OER. As in case of CO_2_ reduction, we can have many products (from formic acid to methane), thus the band gap requirement is product dependent. According to eqn (2)–(6), the thermodynamic potential difference between the oxidation potential and the corresponding reduction potential of CO_2_ are 1.43, 1.35, 1.30, 1.20, and 1.06 eV respectively. It can be seen that the CBM of all the studied systems lies above the potential required to carry out the half reduction reaction. The HSE06 calculated band gap values show that: (i) WS_2_ and WSe_2_ are capable of reducing CO_2_ into all the products, (ii) MoSe_2_ is capable of reducing CO_2_ into HCHO, CH_3_OH, and CH_4_, (iii) MoS_2_ is capable of reducing CO_2_ into methanol and methane. Thus, among the considered systems, WS_2_ and WSe_2_ are the most promising candidates for the photocatalytic reduction of CO_2_ and are capable of performing HER in water splitting, while a co-catalyst would be required to assist the OER. As a final remark, we would like to stress the following. A common first-level criterion to identify potential photocatalytic material in the computational materials-discovery literature is the thermodynamic band-edge alignment against the relavent redox potential,^8,101^ without full Gibbs free energy or charge state calculations, and is used in several first-principles investigations.^102–106^ A complete kinetic and mechanistic description of HER, OER, and CO_2_ reduction require adsorption free-energy calculations of reaction barriers of specific intermediates which is beyond the scope of present screening-level study, and represents a natural direction for future work.

## 4 Conclusions

We have systematically examined the effect of chalcogen vacancies on the structural, optoelectronic properties and photocatalytic water splitting activity in MX_2_ monolayers. Vacancy concentration realising the lowest energy in the convex-hull of MoS_2_, MoSe_2_, and WSe_2_ monolayer is 6.25%, while for WS_2_ it is 1.56%; such concentrations have been considered for the present study. The structural analysis revealed that M–X bond lengths about the vacancies are very close to those found in the pristine structure, this suggesting the absence of local strains which might affect the stability of the atom geometry. The thermal and dynamical stability of all the structures has been examined by performing *ab initio* molecular dynamic simulations and phonon band structure calculations, which showed that all the structures are thermally and dynamically stable. The introduction of vacancies resulted in the introduction of electronic states within the band gap, with a consequent reduction of the gap width. The calculated projected band structures showed that the major contribution to both VBM and CBM come from the d-orbitals of the transition metal, while some minor contribution from the chalcogen atoms can also be observed in some cases. The planar average electrostatic potential analysis revealed that there is a difference of potential between the X layer bearing vacancies and the pristine X layer, with a work function higher on the vacancy side; this may favours charge separation across the layer. Optical absorption coefficient calculations showed that introduction of vacancies resulted in a red-shift of the first absorption peak, while the calculated band edge positions showed that the monolayers are capable of performing hydrogen evolution reaction. Among the considered structures, only WS_2_ and WSe_2_ satisfy the conditions of reducing CO_2_ into useful products, due to favorable conduction band edge positions and band gap which can absorb visible light efficiently; at the same time, all the structures are capable of performing HER in water splitting, while a co-catalyst would be required to assite the OER. These results might constitute guidelines for the experimental synthesis of vacancy-engineered MX_2_ monolayers for optoelectronic devices and photocatalytic applications.

## Conflicts of interest

There are no conflicts to declare.

## Supplementary Material

RA-OLF-D6RA08882A-s001

## Data Availability

Data for this article, including relaxed geometries and input simulation parameters are available at Zenodo at https://doi.org/10.5281/zenodo.21142685. Supplementary information (SI): optimized geometries, *Ab initio* molecular dynamics simulations, electronic band structures. See DOI: https://doi.org/10.1039/d6ra08882a.
