# Supplementary material for: First principles study of chalcogen vacancy effect on the optoelectronic and photocatalytic properties of transition metal dichalcogenides monolayers
Source: RSC Adv. 2026 Nov 3. Online ahead of print. doi: 10.1039/d6ra08882a (PMC13730537; doi:10.1039/d6ra08882a)
Supplement: Supplementary file 1 [file RA-OLF-D6RA08882A-s001.pdf]

Supplementary Information (SI) for RSC Advances.
This journal is © The Royal Society of Chemistry

Journal Name

ARTICLE TYPE

Cite this: DOI: 00.0000/xxxxxxxxxx

# First principles study of chalcogen vacancy effect on the optoelectronic and photocatalytic properties of transition metal dichalcogenides monolayers — Electronic Supplementary Information

Tahir Wahab,*[a] Antonio Cammarata*[a] and Tomas Polcar[b]

## Contents

1 Optimised Structures 2
1.1 $MoS_2$ . . . 2
1.2 $MoSe_2$ . . . 3
1.3 $WS_2$ . . . 5
1.4 $WSe_2$ . . . 7

2 Ab Initio Molecular Dynamics Simulations 8

3 Electronic Band Structures 11

[a] *Department of Control Engineering, Faculty of Electrical Engineering, Czech Technical University in Prague, Technicka 2, 16627 Prague 6, Czech Republic. Fax: +420 224 91 8646; Tel: +420 224 35 5713; E-mail: wahabtah@fel.cvut.cz, cammaant@fel.cvut.cz*
[b] *Engineering Materials & nCATS, FEE, University of Southampton, SO17 1BJ, Southampton, United Kingdom.*

# 1 Optimised Structures

The optimised geometry of each considered defective structures are reported in what follows in POSCAR format.

## 1.1 $MoS_2$

```
MoS
1.00000000000000
12.6213897294682607   -0.0000259318422720    0.0000000000000000
3.1397230101016702   16.4168447338327681    0.0000000000000000
0.0000000000000000    0.0000000000000000   77.8784713744999948
Mo               S
24    45
Direct
0.6381915668048963    0.4472176258087801    0.1154978276738271
0.3879831622785905    0.4482202573929868    0.1151335312912963
0.7240408282357421    0.6102456057040295    0.1152536479419611
0.4707393065130828    0.6102917393707115    0.1151990787773246
0.8037002540097056    0.7759658923354937    0.1161001438843104
0.5553677600061502    0.7787748094299961    0.1157411889927530
0.8883780873257368    0.9430578417801769    0.1176912830783344
0.6357616284828275    0.9420596227607471    0.1164130401322844
0.1383441921989982    0.4470214968377440    0.1154267911009791
0.8878578330488175    0.4483483882739672    0.1153905199262023
0.2256026188924024    0.6096514353716914    0.1151714765099151
0.9698846614305978    0.6096260057748850    0.1152292805415441
0.3084224828078556    0.7759889572444961    0.1160717780369970
0.0560953289846799    0.7758489856821649    0.1169251570848756
0.3932394889697233    0.9420407947002846    0.1163852628027194
0.1401798210321549    0.9430188527964881    0.1176762527112316
0.4727304842042441    0.1093641986381338    0.1170339990663761
0.2229735002387054    0.1106167167436304    0.1176588618472190
0.5551834606481029    0.2790084372848006    0.1168773861609923
0.3055303206869491    0.2790234547431651    0.1168458713236001
0.9722337811758628    0.1109577050358843    0.1180224592319100
0.7217008686885324    0.1106170336970462    0.1177163518511617
0.0551811703852446    0.2791716466096952    0.1171483656812860
0.8052354794167405    0.2791665339015206    0.1171885263452892
0.5270846113123668    0.3875595719359404    0.0957546446964931
0.2793346346655497    0.3875758897187181    0.0957027720919306
0.6104282020238896    0.5584786738121331    0.0951826912509623
0.3614428284617079    0.5565661217875952    0.0949797814097430
0.5233170461410180    0.3969706758345462    0.1358260745089997
0.2780408941281317    0.3969350609392829    0.1357982721956320
0.6105974653768509    0.5563353823418938    0.1355339580660572
0.6919344766959473    0.7220449623917725    0.0955837941954466
0.4470997617589933    0.7221644673459756    0.0955545196160727
0.7774019477022155    0.8909487505898672    0.0967273987637031
0.5277432101163875    0.8893095475604299    0.0960221519565125
0.6938208371604905    0.7200808870371967    0.1358622156798116
0.4460809553153340    0.7200611305438778    0.1358431032092020
0.7707954854784714    0.8847083477265861    0.1368252058746254
```

```
0.0261747791022162    0.3872558931865285    0.0959269791444301
0.7797832211617468    0.3872313920982512    0.0959739280552287
0.1103148674294485    0.5585223029562529    0.0951296229120836
0.8603538470426085    0.5561997978725133    0.0951234962743317
0.0241984561632639    0.3973796642563908    0.1359945453605632
0.7772382521442851    0.3974746133887387    0.1360421586615634
0.1112788754071752    0.5561469051716247    0.1353859408897906
0.0287535831436759    0.8848738171986200    0.1376979940468379
0.1927216915402342    0.7227264404000494    0.0960312703238716
0.9457696563646113    0.7226740450113117    0.0960534991022182
0.2771445773457297    0.8909160076760371    0.0966991802161384
0.0275593267828217    0.8895119705767883    0.0974994738975313
0.2017762842582871    0.7157338802487682    0.1361348726313338
0.9404232224763349    0.7157413030422306    0.1361784916194940
0.2868162644485207    0.8847081951177276    0.1368057262771261
0.1949379021558241    0.2248766851621920    0.1373681825747615
0.3596560907348917    0.0547083452108542    0.0969907190331955
0.1104622988030401    0.0551541180918419    0.0978698979957750
0.4447835261564786    0.2214739104736222    0.0970120586606090
0.1939871743620730    0.2210172450621505    0.0972486406421703
0.3662404673415247    0.0503267679865160    0.1371367049433844
0.1127037859046699    0.0544338911590566    0.1379536795127324
0.4442422791978752    0.2229119064268757    0.1369800755511088
0.6925299171933968    0.2248715086381360    0.1374329644142069
0.8616652375624191    0.0551636634935937    0.0978986442791841
0.6130673849694405    0.0547437115508767    0.0970085648171348
0.9446546810999623    0.2207752377006795    0.0975340517214757
0.6955391676397497    0.2209487893709913    0.0972845929880397
0.8601119137974530    0.0544220021341886    0.1379920974108864
0.6083475706809844    0.0502462214735926    0.1371852050692307
0.9437546157855585    0.2251220173786499    0.1376130644640017
```

### 1.2 $MoSe_2$

```
MoSe
1.00000000000000
13.0828445326117890   -0.0000740622595253    0.0000000000000000
3.2742829995161151   17.0539676515872181    0.0000000000000000
0.0000000000000000    0.0000000000000000   80.0000000000000000
Mo               Se
24    45
Direct
0.6075266212671118    0.4719198226246434    0.2580376475542373
0.3681597944698114    0.4708915900203315    0.2582742875692700
0.6944799302636935    0.6372308009547576    0.2583472744765249
0.4492525769414048    0.6325303966350095    0.2581854974859700
0.7747904593862343    0.8048081294332687    0.2582139029001443
0.5350357196101433    0.8045365741637539    0.2580098413099396
0.8619766726587471    0.9683508159430971    0.2593115169028251
0.6141513157093680    0.9658193960952409    0.2581506844292047
0.1159761504330790    0.4684708947805480    0.2597427634638393
```

| | | |
|---|---|---|
| 0.8623777824689820 | 0.4697175908009962 | 0.2595845900698775 |
| 0.2001655233899039 | 0.6349717517008295 | 0.2594273871681328 |
| 0.9465074486484149 | 0.6362497619644905 | 0.2596747715593321 |
| 0.2836082650208853 | 0.8023022811797931 | 0.2596039507864538 |
| 0.0305414936518494 | 0.8028000743270778 | 0.2597999378947775 |
| 0.3665293246096286 | 0.9704829872052630 | 0.2584438516315959 |
| 0.1155345247158016 | 0.9696784614290133 | 0.2596800506641233 |
| 0.4418121662306360 | 0.1380027215012698 | 0.2580572263041538 |
| 0.2022349388094036 | 0.1382437822833254 | 0.2582534722990529 |
| 0.5273595615817047 | 0.3037885905198041 | 0.2584463134129353 |
| 0.2820749762990024 | 0.2990435371427070 | 0.2582290130271651 |
| 0.9474209749234582 | 0.1360334493084904 | 0.2597468938985720 |
| 0.6944868823476414 | 0.1354615135164810 | 0.2595518373208193 |
| 0.0326889156004502 | 0.3017040356107194 | 0.2593145539639832 |
| 0.7785707552621542 | 0.3029919032830103 | 0.2596100688394087 |
| 0.6428036491935276 | 0.3587824215468836 | 0.2378164841425567 |
| 0.3882702428920112 | 0.3598634611238569 | 0.2373989743235677 |
| 0.7254628770816158 | 0.5234860325206999 | 0.2377485575367301 |
| 0.4736714642495189 | 0.5254906734726433 | 0.2367130890979399 |
| 0.6328392528790325 | 0.3639683570889921 | 0.2796499460542770 |
| 0.3907894350226667 | 0.3585657495109630 | 0.2793727271297705 |
| 0.7145013752115212 | 0.5271174542912137 | 0.2796076478053480 |
| 0.8107575441348575 | 0.6922474125257725 | 0.2378571031639364 |
| 0.5554067019482252 | 0.6928210181821314 | 0.2372451418155975 |
| 0.8928346745529029 | 0.8573012345086720 | 0.2381760943169506 |
| 0.6421510509588997 | 0.8587856162235171 | 0.2366821346990183 |
| 0.7999909315472808 | 0.6963143058618687 | 0.2796877833753145 |
| 0.5577947389585628 | 0.6927718372268686 | 0.2792099909455938 |
| 0.8831716929417815 | 0.8574181552131470 | 0.2801304697507999 |
| 0.9747568060239414 | 0.5247253647793203 | 0.2807574523272754 |
| 0.1422177386085363 | 0.3589797333696717 | 0.2381879514424075 |
| 0.8910681122044668 | 0.3580875312568747 | 0.2387177555059847 |
| 0.2251089751825318 | 0.5241622098610330 | 0.2382477310226290 |
| 0.9758979311710083 | 0.5244973302199053 | 0.2388604898687474 |
| 0.1487665072942037 | 0.3523649275534229 | 0.2801790654817111 |
| 0.8922526312119017 | 0.3577039097018795 | 0.2804690665500426 |
| 0.2347027250686506 | 0.5237982246831718 | 0.2802801958387585 |
| 0.1443878885350037 | 0.8583717303960405 | 0.2808132492526280 |
| 0.3090881109139294 | 0.6922166098336251 | 0.2382065789849023 |
| 0.0587137238728173 | 0.6916880699376375 | 0.2388336911390885 |
| 0.3921723962068877 | 0.8569585746071382 | 0.2378175389077352 |
| 0.1426233128057993 | 0.8579379016081505 | 0.2388758042180014 |
| 0.3165342961048844 | 0.6863698300299923 | 0.2801687937120838 |
| 0.0601135686099496 | 0.6906912340527541 | 0.2806284834654088 |
| 0.4020813256404763 | 0.8601710101297882 | 0.2797324807677249 |
| 0.4773839061704696 | 0.0262929583436344 | 0.2373116625357697 |
| 0.2232817282265424 | 0.0257145358208358 | 0.2378995671903843 |
| 0.5583128979302165 | 0.1902671962219392 | 0.2378216070405317 |
| 0.3077094436915692 | 0.1922457501863613 | 0.2367060744385512 |
| 0.4758459303631853 | 0.0260750878117148 | 0.2792478813165732 |
| 0.2315396227061713 | 0.0299840065032077 | 0.2797808109827201 |

```
0.5474069377630898    0.1934186392361248    0.2797394035722225
0.8057847605188642    0.1915657067153465    0.2807376980411816
0.9756050778387947    0.0249990796891552    0.2387681009601618
0.7241868894921596    0.0254461940516709    0.2381118091920977
0.0580348036169570    0.1906504970986406    0.2381692013070889
0.8076920106278717    0.1911374004158201    0.2388351970933363
0.9740716628299416    0.0238720425061494    0.2805522909919991
0.7201654593063658    0.0195520682439520    0.2801018278354632
0.0671124035909484    0.1907540174139143    0.2801280029270347
```

### 1.3 $WS_2$

```
WS
1.00000000000000
22.9190387987332791    0.0001746768093215    0.0000000000000000
19.3939147253939872   12.2130421600155454    0.0000000000000000
0.0000000000000000    0.0000000000000000   80.0000000000000000
S                W
63    32
Direct
0.0492181476643486    0.5341621322388966    0.1331617870198417
0.4730928963818157    0.2308993396535041    0.1331059179357131
0.2308993047370883    0.4730928886625529    0.1331059686486036
0.2916237578828197    0.2916239804183076    0.0925602847211653
0.0419011300701079    0.5402854150919786    0.0939470514587715
0.4770276515844877    0.2308026387517758    0.0939100173671708
0.2308025408790316    0.4770277665673519    0.0939100708048253
0.6653521048503833    0.1667088724845747    0.1335543674664848
0.4161535171151112    0.4161535233274515    0.1336311255387171
0.8539009761654682    0.1039734714687173    0.1334582826932089
0.6040880554256438    0.3539859372256441    0.1335219097977258
0.6653425538138020    0.1670946316356008    0.0943486103129746
0.4163107085966875    0.4163109114132675    0.0944067327979460
0.8534823970641431    0.1041612830523746    0.0942918236590053
0.6036607950718919    0.3540762272083687    0.0943362891503482
0.0416608150632711    0.0416614362722312    0.1333918416976596
0.7917998081541479    0.2915875139408782    0.1334135608283055
0.2292083385322204    0.9796378928548656    0.1334275323101162
0.9796375229652355    0.2292086360334186    0.1334275517959843
0.0416346624938281    0.0416347849828281    0.0942297074457215
0.7916391753662739    0.2917243635878691    0.0942386479487579
0.2294807023754685    0.9795120106390496    0.0942509356655686
0.9795120585090314    0.2294807148254268    0.0942509680573640
0.1667085155302827    0.6653524481483533    0.1335544086332547
0.9162297297119192    0.9162303288468659    0.1334584980274021
0.3539858051784397    0.6040882226238578    0.1335219200619509
0.1039730036805400    0.8539014499120421    0.1334583007074431
0.1670946531674168    0.6653425134398714    0.0943486697537793
0.9162478781889773    0.9162476860755420    0.0943123788697501
0.3540761245890537    0.6036608698215062    0.0943363023475993
0.1041614143573116    0.8534822228698560    0.0942918582151160
```

| | | |
|---|---|---|
| 0.5417374177572410 | 0.5417377913747690 | 0.1334192967316614 |
| 0.2915870530781620 | 0.7918002580059171 | 0.1334135842149553 |
| 0.7295510127184742 | 0.4791216364168773 | 0.1334674891968694 |
| 0.4791212802035100 | 0.7295514112367292 | 0.1334674923602035 |
| 0.5417199060115052 | 0.5417200409659808 | 0.0942496564419621 |
| 0.2917242063107968 | 0.7916392962432637 | 0.0942386801992743 |
| 0.7296609619975625 | 0.4794685225682691 | 0.0942900518651081 |
| 0.4794685058309538 | 0.7296609663844758 | 0.0942900461057123 |
| 0.9181766879098927 | 0.4162281710341607 | 0.1335879270359372 |
| 0.6668101472116391 | 0.6668105491554586 | 0.1336628838087892 |
| 0.1103518906302444 | 0.3522360175838030 | 0.1331277547410590 |
| 0.8554908976955437 | 0.6034370076945742 | 0.1337498245125641 |
| 0.9172461097645294 | 0.4172193308922130 | 0.0943692590003430 |
| 0.6671282436888745 | 0.6671281148228937 | 0.0944570028213561 |
| 0.1038732890563835 | 0.3558532817143827 | 0.0939269069303212 |
| 0.8542602487234007 | 0.6045095618086654 | 0.0945261745198906 |
| 0.4162278997550062 | 0.9181770015837747 | 0.1335879060846138 |
| 0.1676429838664575 | 0.1676430437549794 | 0.1333420947093015 |
| 0.6034365791294137 | 0.8554913779993755 | 0.1337497796002838 |
| 0.3522360507441716 | 0.1103518409215805 | 0.1331277038330443 |
| 0.4172194200771975 | 0.9172459396339432 | 0.0943692315929897 |
| 0.1670435361690757 | 0.1670435914944811 | 0.0942079979709968 |
| 0.6045097229718757 | 0.8542600655245451 | 0.0945261429684260 |
| 0.3558531238287158 | 0.1038733523514989 | 0.0939268513897335 |
| 0.7916825586480897 | 0.7916832899314479 | 0.1337129855264615 |
| 0.5341617015216715 | 0.0492186130123673 | 0.1331617299843246 |
| 0.9805626933434859 | 0.7266173515076404 | 0.1333831309015717 |
| 0.7266168287836953 | 0.9805632745158229 | 0.1333831023202080 |
| 0.7913819660786221 | 0.7913818247821404 | 0.0944981176274667 |
| 0.5402853747608986 | 0.0419011669041295 | 0.0939469825545464 |
| 0.9787108622995001 | 0.7289346384063030 | 0.0942444616697749 |
| 0.7289347503023814 | 0.9787107466908472 | 0.0942444140072698 |
| 0.1532196313667590 | 0.3907577194190847 | 0.1129619761558572 |
| 0.8971684038663921 | 0.6446959920538994 | 0.1140658075720276 |
| 0.3311417839984316 | 0.3311418064024079 | 0.1129300697463723 |
| 0.0849662458158725 | 0.5806827157190088 | 0.1135445021977462 |
| 0.5192899509910766 | 0.2710874526662100 | 0.1139508742708273 |
| 0.2710873953501118 | 0.5192899875689381 | 0.1139509113017438 |
| 0.7076520887285233 | 0.2082644321597195 | 0.1139373977904272 |
| 0.4580396204263421 | 0.4580396930028233 | 0.1139783124263333 |
| 0.8956713223905171 | 0.1457964554557117 | 0.1138205402384763 |
| 0.6457772346054804 | 0.3958066096507867 | 0.1138438933245749 |
| 0.0835165095622814 | 0.0835165545468608 | 0.1138094006447509 |
| 0.8336492263627101 | 0.3334185296835113 | 0.1138321414484074 |
| 0.0210012542132349 | 0.7698936358463980 | 0.1138957416449165 |
| 0.7698933901082875 | 0.0210014918128500 | 0.1138957194348436 |
| 0.2082643660346918 | 0.7076521716426096 | 0.1139374269627290 |
| 0.9580346052140749 | 0.9580348723169286 | 0.1138764188433136 |
| 0.3958063728444540 | 0.6457774558671475 | 0.1138439176150904 |
| 0.1457961594371140 | 0.8956716460159235 | 0.1138205806325951 |
| 0.5835715901033268 | 0.5835717984577021 | 0.1138431251409575 |

```
0.3334182842027653    0.8336494730234765    0.1138321592003350
0.7716347991135647    0.5208878375501882    0.1139866653127622
0.5208877175157823    0.7716349074691999    0.1139866597197917
0.9599129110501456    0.4580873524203163    0.1140034297930520
0.7086579016027081    0.7086579399607505    0.1141779251930502
0.2711786123446731    0.0214591796861075    0.1138756837730564
0.0214591203449390    0.2711786908838417    0.1138756911079865
0.4580872225331887    0.9599130014657942    0.1140033830880529
0.2093719355666808    0.2093720169287177    0.1135208508274161
0.6446957696052609    0.8971686810301598    0.1140657703946680
0.3907576665244589    0.1532196333712869    0.1129619155790337
0.8330455928779824    0.8330459254101998    0.1139736669698431
0.5806826472629597    0.0849663485193237    0.1135444346836498
```

### 1.4 $WSe_2$

```
WSe
1.00000000000000
13.1206362457420447    0.0014710624231543    0.0000000000000000
3.2782401790727471   17.0711920690310421    0.0000000000000000
0.0000000000000000    0.0000000000000000   80.0000000000000000
Se                W
45    24
Direct
0.5260111118542748    0.3887853233544605    0.1470875540479449
0.2804951171379100    0.3878441205831221    0.1462545809415732
0.6101512517864763    0.5567206401107949    0.1471589801992827
0.3626692203596217    0.5551121837183438    0.1443391255667206
0.5179395058296519    0.3950248275875556    0.1889767253075579
0.2787254361528331    0.3876676643816488    0.1879050445476648
0.5994849296606573    0.5570418489393516    0.1891509940966619
0.6930583839495732    0.7219842127656924    0.1471997775489625
0.4479903432935511    0.7207611565497947    0.1463972509057099
0.7768107479163042    0.8900942894898265    0.1472692225348904
0.5281334203522583    0.8882660663195833    0.1443784124392487
0.6857195576086205    0.7289281984422500    0.1890820409670624
0.4452509009830337    0.7220219182023304    0.1880651730238952
0.7677084191185958    0.8900260167462629    0.1890434417094702
0.8614254509219265    0.5558706153327162    0.1897095571270613
0.0258532523621050    0.3873829786339733    0.1468858991059975
0.7773176714986421    0.3887249927745221    0.1476873307068865
0.1117727824783408    0.5572533819471048    0.1467299477231727
0.8604099516511892    0.5559342730327419    0.1478678617798382
0.0369296775004939    0.3834562646809850    0.1886543085999941
0.7753458723306841    0.3899780773566009    0.1896549096589923
0.1230902935710870    0.5536062554984741    0.1885526267195292
0.0313130821796565    0.8889859460961934    0.1896324645980866
0.1939453430807492    0.7208410693576609    0.1467344450453786
0.9449274383472827    0.7224559812501504    0.1475701705971893
0.2786406538364097    0.8905225401329958    0.1466079584473551
0.0286541004244584    0.8894265294101649    0.1477841318065151
```

| | | |
|---|---|---|
| 0.2043530888267712 | 0.7158195662994765 | 0.1885193190861408 |
| 0.9442212461212821 | 0.7229497724522950 | 0.1895161165880536 |
| 0.2902374478247463 | 0.8874078917025507 | 0.1886253915851938 |
| 0.3587381829400821 | 0.0543500918195786 | 0.1462693991536969 |
| 0.1125763220167996 | 0.0557002225282651 | 0.1470561139924965 |
| 0.4429653153396975 | 0.2238199441173839 | 0.1466185248835093 |
| 0.1942412539590694 | 0.2219151699354390 | 0.1443428207042262 |
| 0.3602430477830280 | 0.0544304374583200 | 0.1879056654886850 |
| 0.1174658263527650 | 0.0617496155312349 | 0.1889337334903210 |
| 0.4323627192400898 | 0.2206392362133604 | 0.1886333488865216 |
| 0.6907139348103076 | 0.2223347781251638 | 0.1897317166316708 |
| 0.8606006282352060 | 0.0555200101499569 | 0.1476438782428526 |
| 0.6128592150543057 | 0.0538118650335428 | 0.1468884232084021 |
| 0.9445088040406298 | 0.2234760181284364 | 0.1472632672200009 |
| 0.6932429180835079 | 0.2224404256238566 | 0.1478504433427303 |
| 0.8620960339838523 | 0.0567184662739627 | 0.1896390390750409 |
| 0.6037859911845281 | 0.0499006208721042 | 0.1887018185457981 |
| 0.9541489328617769 | 0.2232694500706827 | 0.1890425962231820 |
| 0.6355668834042337 | 0.4456490045644966 | 0.1682864523498774 |
| 0.3856743953733158 | 0.4522212696316164 | 0.1669260208522374 |
| 0.7193065386479203 | 0.6121908735012151 | 0.1687104113487428 |
| 0.4638742252393719 | 0.6075694783035994 | 0.1670241926048402 |
| 0.8034728337987016 | 0.7786771482498137 | 0.1682877658347822 |
| 0.5526456405555528 | 0.7857276794921259 | 0.1670336549299956 |
| 0.8884851224132722 | 0.9447188099612759 | 0.1687429749707608 |
| 0.6287475060483040 | 0.9398839329785259 | 0.1671561746771356 |
| 0.1413263486614121 | 0.4434411758565412 | 0.1672584881570094 |
| 0.8896796294290915 | 0.4436870589418013 | 0.1686673712050331 |
| 0.2346329746384886 | 0.6061710128405728 | 0.1668749954646570 |
| 0.9744689292926364 | 0.6110138750889926 | 0.1685603978994538 |
| 0.3085107853631789 | 0.7770608369749928 | 0.1672590124432105 |
| 0.0579665190865066 | 0.7769774365003292 | 0.1685282310459562 |
| 0.3990328182121907 | 0.9403965441822093 | 0.1667852330602325 |
| 0.1429443637621692 | 0.9452610889431388 | 0.1684290488907679 |
| 0.4697175177202651 | 0.1103557769616294 | 0.1672717258277776 |
| 0.2214310201419362 | 0.1189628159775616 | 0.1669072980547444 |
| 0.5511140984215626 | 0.2784313148304351 | 0.1684863728888808 |
| 0.2973956585909237 | 0.2738284819955734 | 0.1667554802618804 |
| 0.9742415925672658 | 0.1120200235986724 | 0.1682612753911408 |
| 0.7206664398347500 | 0.1102405455039421 | 0.1686645706365235 |
| 0.0676325233793015 | 0.2733618107133940 | 0.1671207271876926 |
| 0.8056688615728227 | 0.2778500003765962 | 0.1687865619154863 |

## 2 Ab Initio Molecular Dynamics Simulations

Temperature and total energy as a function of the time are reported in Figure S1 and Figure S2, respectively.

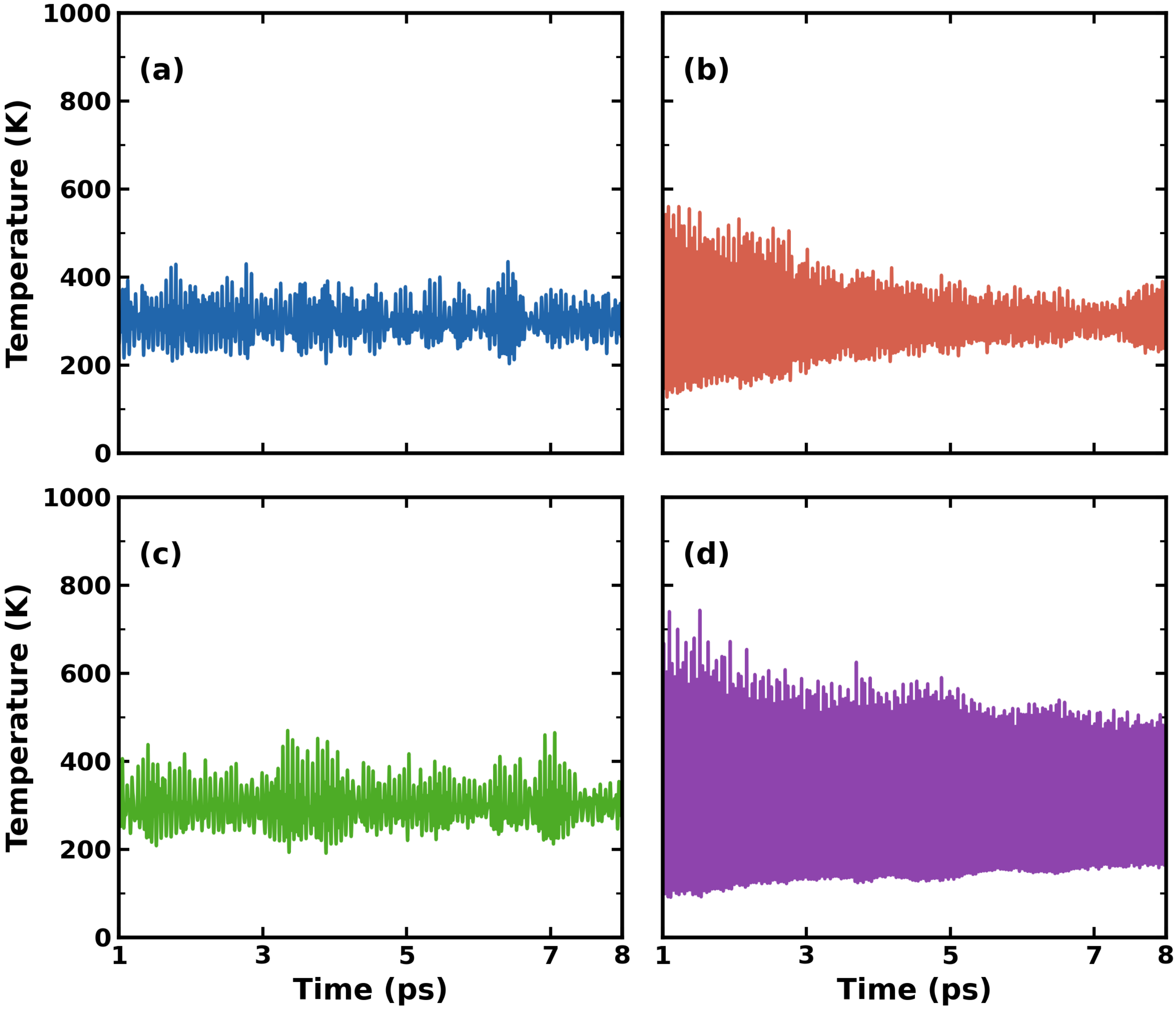


**Fig. S1** Temperature as a function of the simulation time of the (a) $MoS_2$, (b) $MoSe_2$, (c) $WS_2$, and (d) $WSe_2$ systems.

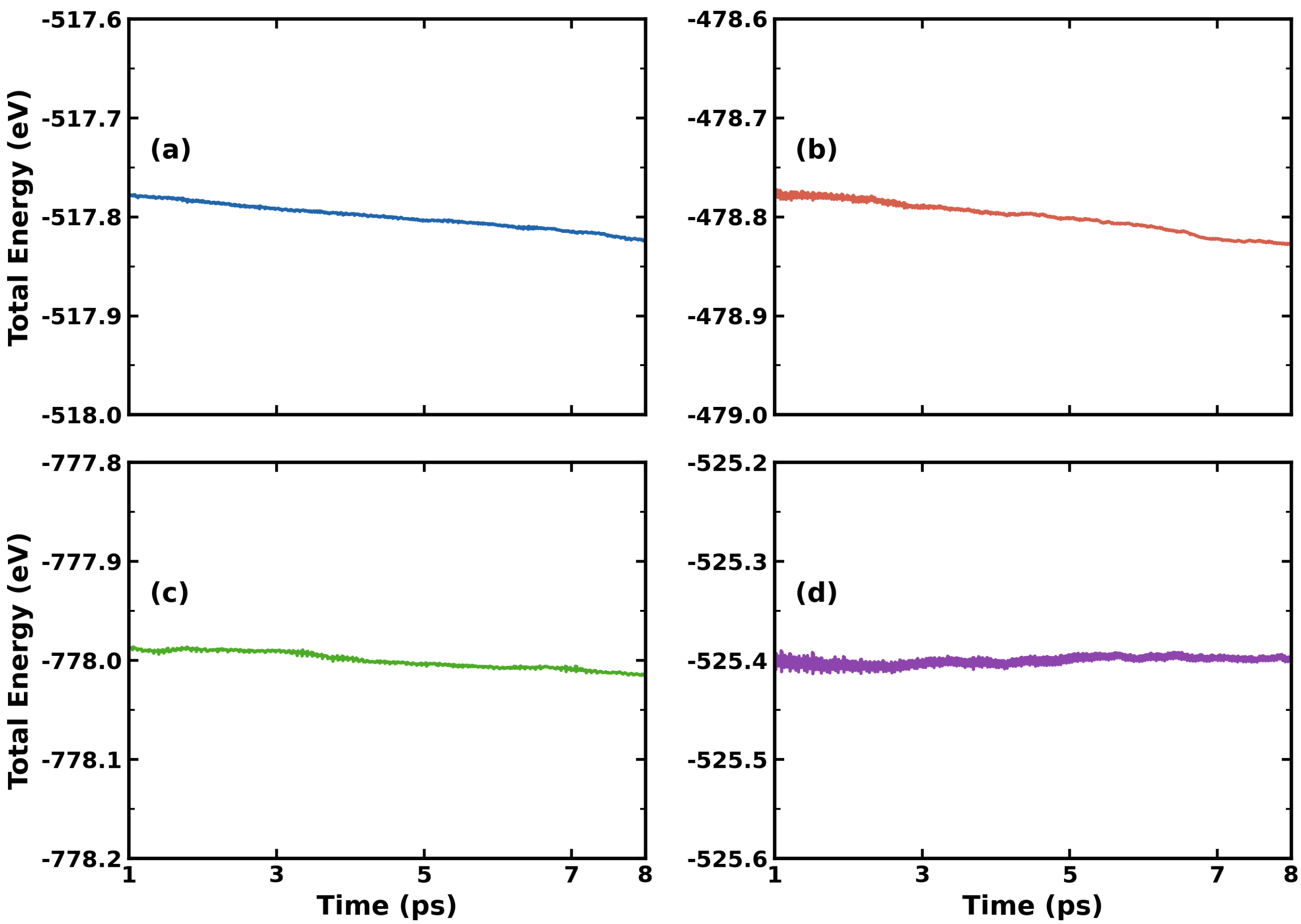


**Fig. S2** Total energy as a function of the simulation time of the (a) $MoS_2$, (b) $MoSe_2$, (c) $WS_2$, and (d) $WSe_2$ systems.

## 3 Electronic Band Structures

In Figure S3, we report the electronic band structure of the selected systems calculated using the PBE energy functional. Plots reported in Figure S4 show the shift induced by the spin-orbit correction to the electronic band structures calculated with the PBE energy functional. Figure S5 shows the spin-projected electronic band structure using the PBE energy functional of the selected system.

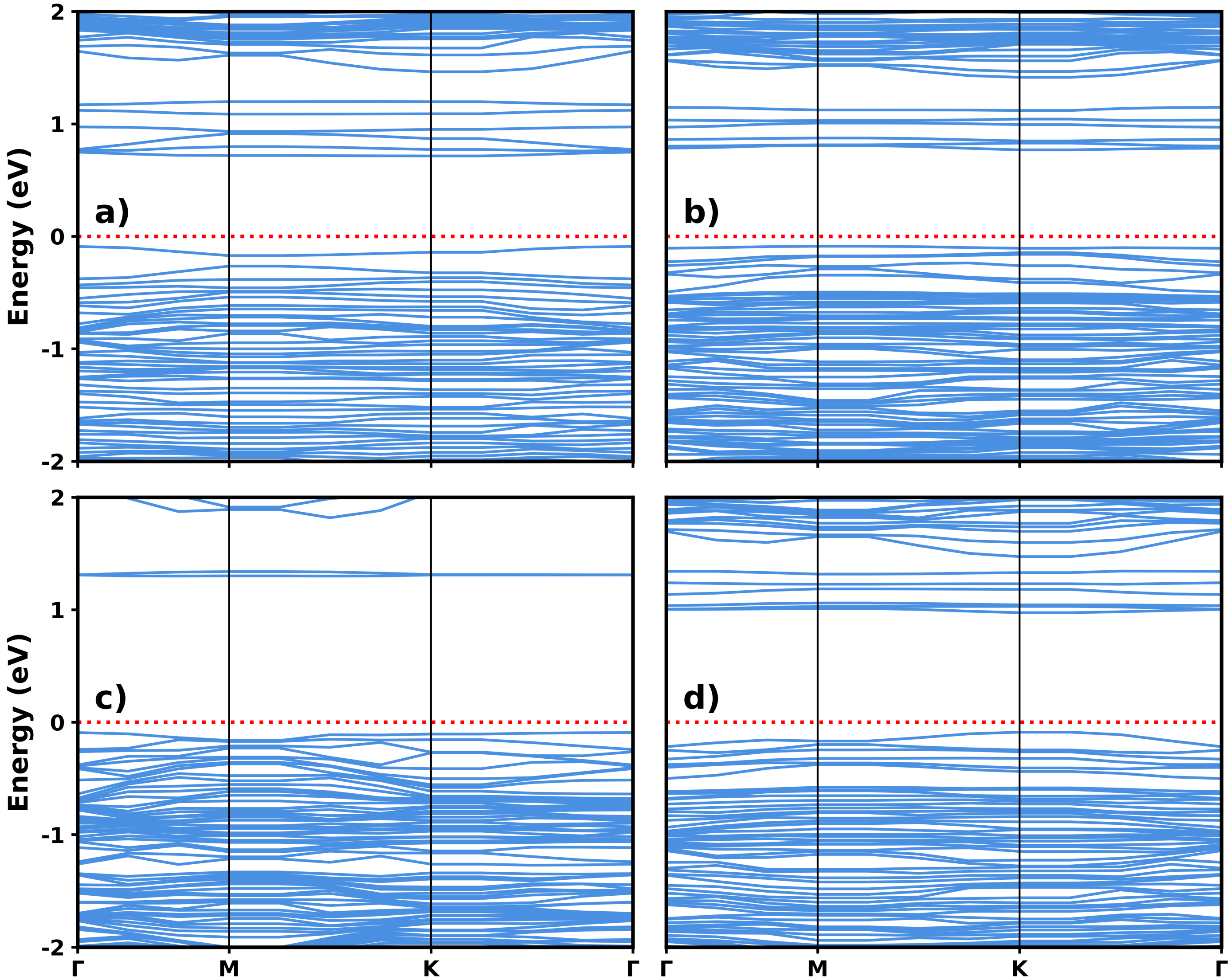


**Fig. S3** Electronic band structure of (a) $MoS_2$, (b) $MoSe_2$, (c) $WS_2$, and (d) $WSe_2$ defective systems calculated along the high symmetry path by using the PBE energy functional. The Fermi level has been set to 0 eV and is indicated by a dashed line.

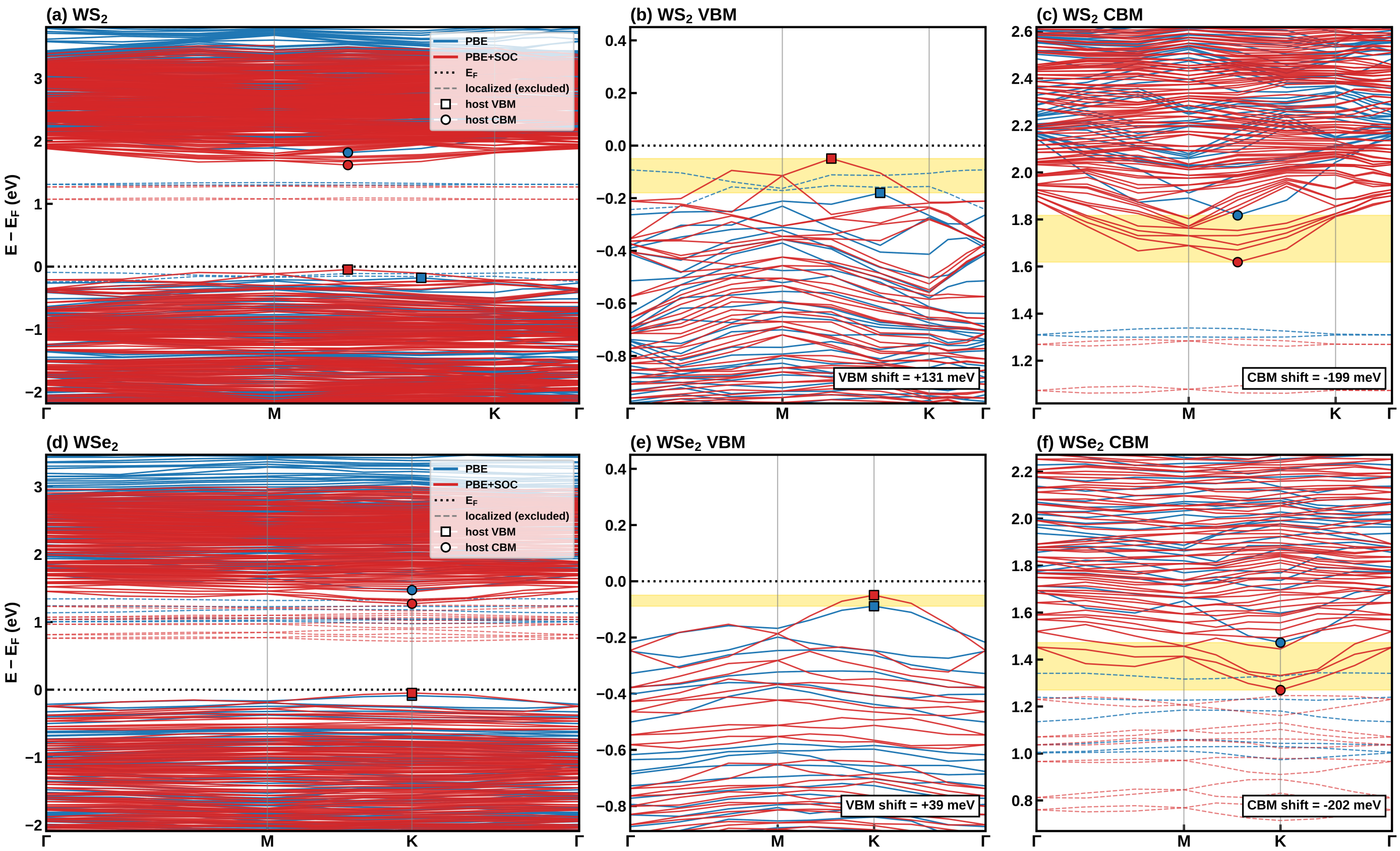


**Fig. S4** Electronic band structure calculated with PBE (blue lines) and PBE+SOC (red lines) energy functionals of (a) $WS_2$, and (d) $WSe_2$ defective systems calculated along the selected high symmetry path. The fermi level is indicated by a dotted black line. The detail of the corresponding band edges is reported in (b), (c), (e) and (f) subfigures, as labeled on top of each plot; coloured squares and circles indicate the position of the valence band maxima and conduction band minima, respectively.

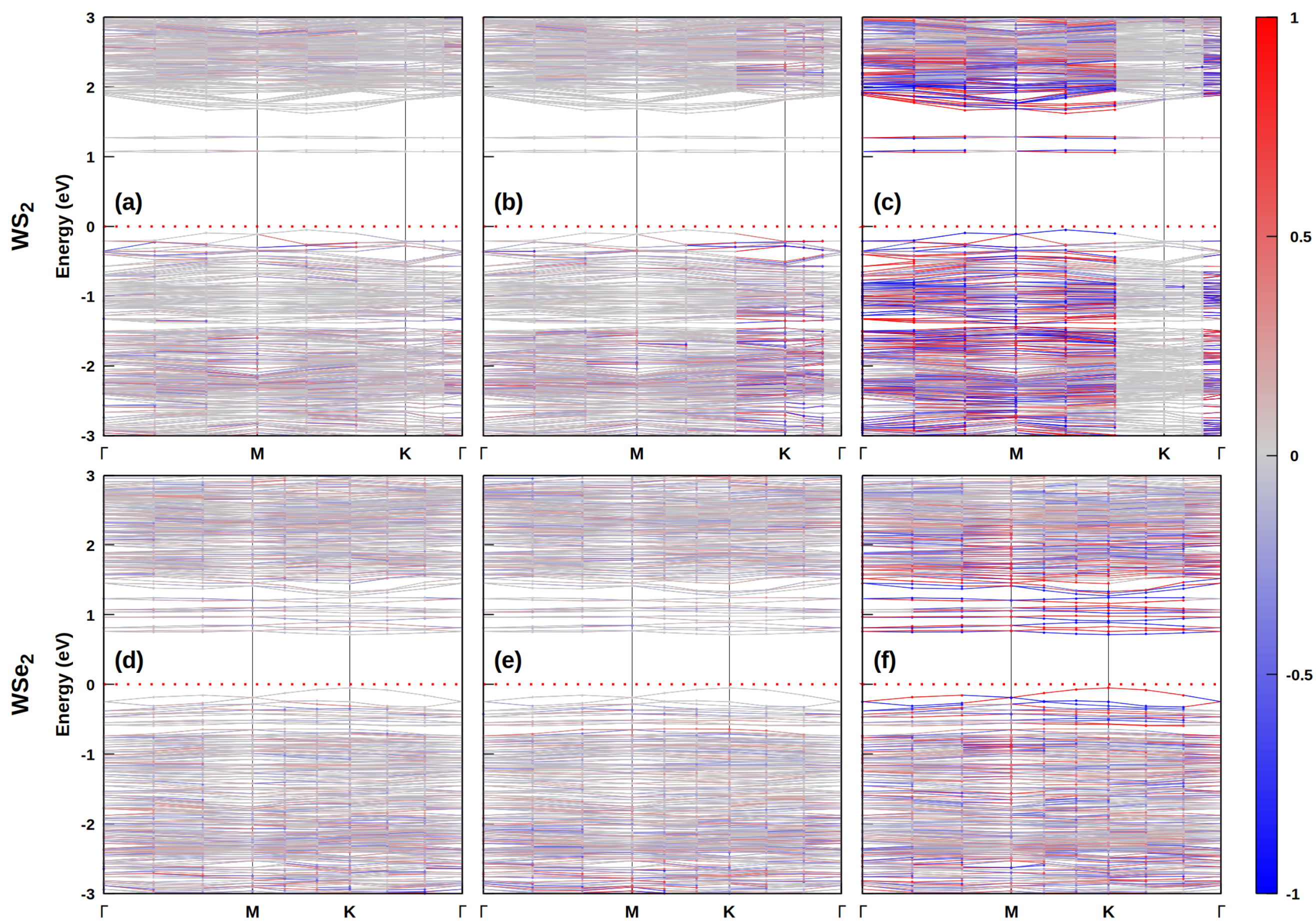


**Fig. S5** Spin-projected band structure calculated with PBE+SOC energy functional of (a-c) $WS_2$, and (d-f) $WSe_2$ defective systems calculated along the selected high symmetry path. The Fermi level is indicated by a dotted red line. Panels (a-c) shows the $S_x$, $S_y$, and $S_z$ component of $WS_2$ respectively, while panels (d-f) shows the $S_x$, $S_y$, and $S_z$ component of $WSe_2$ respectively. The color scale gives the spin expectation values of each band, where red and blue corresponds to +1 and -1, respectively.
